# Engineered red blood cells (activating antigen carriers) drive potent T cell responses and tumor regression in mice

**DOI:** 10.3389/fimmu.2022.1015585

**Published:** 2022-10-03

**Authors:** Katarina Blagovic, Carolyne K. Smith, Amritha Ramakrishnan, Lindsay Moore, David R. Soto, Zachary Thompson, Adam P. Stockmann, Sonia Kruszelnicki, Akshi Thakkar, Jason Murray, Sebastian Torres, Bersabel Wondimagegnhu, Roslyn Yi, Maisam Dadgar, Abdul M. Paracha, Claire Page, Louise Clear, Omer A. Chaudhry, Melissa Myint, Devin T. Bridgen, Jonathan B. Gilbert, Katherine J. Seidl, Armon Sharei, Scott Loughhead, Howard Bernstein, Defne Yarar

**Affiliations:** SQZ Biotechnologies Company, Watertown, MA, United States

**Keywords:** CD8 T cells, dendritic cells, red blood cells, adjuvant, activating antigen carriers, antigen presenting cell, cancer immunotherapy, human papillomavirus

## Abstract

Activation of T cell responses is essential for effective tumor clearance; however, inducing targeted, potent antigen presentation to stimulate T cell responses remains challenging. We generated Activating Antigen Carriers (AACs) by engineering red blood cells (RBCs) to encapsulate relevant tumor antigens and the adjuvant polyinosinic-polycytidylic acid (poly I:C), for use as a tumor-specific cancer vaccine. The processing method and conditions used to create the AACs promote phosphatidylserine exposure on RBCs and thus harness the natural process of aged RBC clearance to enable targeting of the AACs to endogenous professional antigen presenting cells (APCs) without the use of chemicals or viral vectors. AAC uptake, antigen processing, and presentation by APCs drive antigen-specific activation of T cells, both in mouse *in vivo* and human *in vitro* systems, promoting polyfunctionality of CD8+ T cells and, in a tumor model, driving high levels of antigen-specific CD8+ T cell infiltration and tumor killing. The efficacy of AAC therapy was further enhanced by combination with the chemotherapeutic agent Cisplatin. In summary, these findings support AACs as a potential vector-free immunotherapy strategy to enable potent antigen presentation and T cell stimulation by endogenous APCs with broad therapeutic potential.

## Introduction

Immune checkpoint inhibitors have shown efficacy in the clinic, but success has been limited primarily to individuals with existing CD8^+^ T cell responses (1–3). Therefore, there has been significant interest in using therapeutic vaccines to generate tumor-targeting CD8^+^ T cell responses. A significant challenge to this approach is to find a therapy which specifically delivers tumor antigens and adjuvants to antigen presenting cells (APCs) in a format that ensures all three signals for T cell activation are engaged: 1. Peptide-MHC, 2. Co-stimulation, 3. Inflammatory cytokines (2).

Different therapies aim to target one or more of three activation signals. Dendritic cell (DC)-based vaccines, for example, are generated by incubating DCs with tumor antigens *ex vivo*, but these strategies typically require considerable manufacturing times, produce a heterogenous mixture of DC subsets, and/or have challenges homing to the lymphoid organs for effective T cell priming (4, 5). Additional alternative strategies include nanoparticles and viral vectors that seek to target endogenous APCs. However, these use non-natural components or infectious agents with suboptimal targeting of APCs, potentially eliciting unintended immunological responses, and thus leading to adverse events or neutralization of the drug product (6–8). Here, we demonstrate that *in vivo* delivery of tumor antigens and adjuvant to APCs can be accomplished by engineering red blood cells (RBCs) to generate an effective cancer vaccine – leveraging a physiological targeting mechanism that could avoid the pitfalls of the aforementioned therapies.

RBCs have been explored as drug carriers, but their mechanism of action (MOA) has mainly been limited to delivering or entrapping materials within the blood stream, such as with enzyme replacement therapies (9, 10). In the context of RBC-based cancer therapies (11–14), while the MOA is more systemic, the technology often necessitates difficult dialysis strategies for antigen loading, or chemical modifications of the RBC membrane. Using a microfluidic chip system previously described for payload delivery to peripheral blood mononuclear cells (PBMCs) (15–17), we encapsulate peptide or protein antigens and adjuvant into RBCs for therapeutic applications, which we term Activating Antigen Carriers (AACs). As a result of processing RBCs using this technology, an increase in surface phosphatidylserine (PS) and cell shrinkage are induced. These are natural markers of eryptosis (18–23) that lead to the rapid uptake of the processed RBCs by APCs in the reticuloendothelial system (RES) following intravenous administration.

By encapsulating immunogenic materials (antigens and adjuvant) within the engineered RBC carriers, we protect the cargo from undesired degradation, anti-viral vector humoral immunity, and attempt to avoid systemic inflammation, as compared to administration of free antigen and/or adjuvant (2, 24, 25). In this study, we will show that, after intravenous administration, the AACs are taken up in the spleen and the liver. The immunogenic cargo then promotes localized APC activation and maturation of toll-like receptors (TLR)-sensitive cell types, further avoiding non-specific inflammation and instead supplying T cells with all 3 signals needed for full activation (4–8).

We show that the activation promoted by AACs is dependent on the presence of the antigen and the adjuvant poly I:C. These antigen-specific responses are seen both in CD8^+^ and CD4^+^ T cells after *in vivo* AAC administration to mice, indicating cross-presentation of the antigen by endogenous APCs. Finally, in a mouse model of human papillomavirus (HPV) HPV16^+^ tumors (TC-1), therapeutic treatment with AACs significantly slowed tumor growth, and increased tumor infiltration of antigen-specific CD8^+^ T cells. Therapeutic treatment with AACs also showed enhanced efficacy when combined with the chemotherapeutic agent, Cisplatin. Our study shows that engineering of RBCs with cancer antigens and adjuvant can leverage the natural process of aged RBC uptake and represents a promising new therapeutic approach to cancer treatments.

## Results

### Engineered RBCs are loaded with antigens and are rapidly cleared from circulation

**Figure 1:**
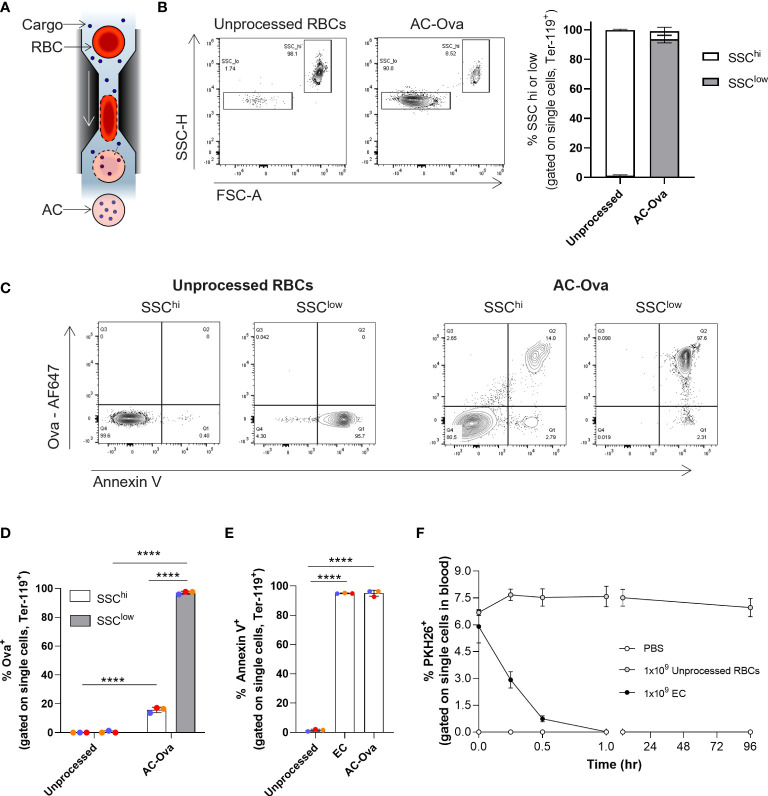
Cell Squeeze^®^ platform generates Antigen Carriers (AC) which exhibit rapid *in vivo* uptake. **(A)** Schematic of Cell Squeeze^®^ microfluidic platform for intracellular delivery of cargo to RBCs. **(B)** Unprocessed mouse RBCs and Ova-AF647 squeezed carriers (AC-Ova) with distinct SSC^low^ and SSC^hi^ populations (from single cell gate). Left: flow plots (SSC-H vs. FSC-A). Right: Percent SSC^low^ and SSC^hi^ populations. **(C)** Annexin V levels and Ova-AF647 delivery in unprocessed RBCs and AC-Ova. **(D)** Percent Ova-AF647 delivery to events in SSC^low^ and SSC^hi^ populations. **(E)** Percent annexin V positive events in unprocessed RBC or carrier groups. **(F)** *In vivo* clearance kinetics of untouched RBCs and EC from n = 2 mice in PBS group and n = 3 mice in each unprocessed RBC and EC group. n = 2 independent studies. ****P < 0.0001, one-way ANOVA.

The Cell Squeeze^®^ platform (**Figure 1A**) has been used to deliver a variety of macromolecules to diverse mononucleated cell types as previously described (15–17). We use this technology to engineer RBC carriers for use in therapeutic applications. Murine RBCs were isolated from mice and squeezed through the microfluidic device with media alone, referred to as empty carriers (EC), or in the presence of antigen (fluorescently labeled Alexa Fluor 647 ovalbumin, Ova-AF647), referred to as antigen carriers (AC-Ova). We examined different microfluidic chip designs and process parameters to identify conditions that resulted in the highest frequency of Ova-AF647^+^ carriers with characteristics of aged RBCs, such as an increase in PS levels and a drop in FSC, that would encourage uptake by APCs (18). The overall cell yields relative to input were 24% to 50% at the optimized squeeze conditions.

By flow cytometry, we found that engineered RBCs took on a distinct low side scatter SSC-H and FSC-A profile (referred to as SSC^low^) compared to unprocessed RBCs which exhibited high SSC-H and FSC-A intensities (referred to as SSC^hi^) (**Figure 1B**; **Figure S1A**) (18). Both squeeze processed and unprocessed RBCs stained positive for the mouse erythrocyte marker, Ter-119 (**Figure S1A**) (26). Generation of the SSC^low^ population was process dependent as both EC and AC-Ova had similar flow characteristics (**Figure S1A**). Further flow cytometry analysis of AC-Ova revealed that only a small fraction of SSC^hi^ cells was positive for the Ova-AF647 signal, while the majority of the SSC^low^ cells were positive for Ova-AF647 (**Figures 1C, D**, 15.8% ± 1.9% vs 97.1% ± 1.0%, n = 3 independent experiments). In summary, the engineering of the RBCs generated carriers positive for delivery material that exhibited characteristics resembling that of aged RBCs.

As mentioned, the display of PS is a hallmark of aged RBCs that we sought to characterize on our carriers. The surface exposure of PS, which normally exists in the inner leaflet of the plasma membrane, is recognized as an “eat-me” signal for the uptake of damaged, aged cells, ensuring their clearance by APCs (18, 20, 27, 28). We stained unprocessed RBCs, EC and AC-Ova with annexin V, a PS binding protein, and analyzed the cells using flow cytometry. The frequency of cells positive for surface exposed PS was significantly elevated in processed samples relative to unprocessed RBCs (unprocessed RBCs 1.5% ± 0.8%, EC 95.0% ± 0.2%, AC-Ova 95.1% ± 2.1%, n=3 independent experiments, **Figure 1E**), and was significantly higher on SSC^low^ compared to SSC^hi^ cells (**Figure S1A**). Because annexin V levels were similar between EC and AC-Ova, the observed changes in annexin V levels are mediated by the Cell Squeeze processing and independent of delivered materials.

To examine circulation kinetics of the engineered RBCs, we intravenously injected EC or unprocessed RBCs, each labeled with the fluorescent, lipophilic membrane dye PKH26, into mice and quantified the frequency of carriers or RBCs in the blood over the course of multiple days (up to 96 hours post injection) using flow cytometry. In contrast to unprocessed RBCs, which were maintained in circulation throughout the time course without a significant change in frequency, carriers were rapidly removed from the blood with a half-life of 12.8 ± 2.1 minutes (**Figure 1F**) and were undetectable in the bloodstream 1 hour following administration, similar to aged and senescent RBCs, reported to be rapidly cleared by APCs (20, 27, 29, 30). Others have shown that this trait can be utilized to promote targeted delivery of cancer antigens to the immune system to induce anti-cancer responses (11, 14, 31, 32).

ACs have properties similar to aged RBCs in that they are rapidly cleared from circulation and have surface membrane exposed PS. Additionally, we examined the potential decrease in the levels of the “don’t-eat-me” signal CD47, reported as relevant in the uptake of aged or apoptotic cells (20, 22, 33, 34), however, in our studies no significant changes were observed (**Figure S1B**). Nonetheless, the dramatic differences observed in the clearance kinetics of different RBC groups correlate directly with differences observed in physical properties reflected in flow cytometry light scattering, and annexin V levels. To demonstrate that these carriers can be processed by and activate the immune system, *in vivo* mouse studies were performed.

### AACs prime endogenous CD8^+^ T cell responses to protein and peptide antigens

**Figure 2:**
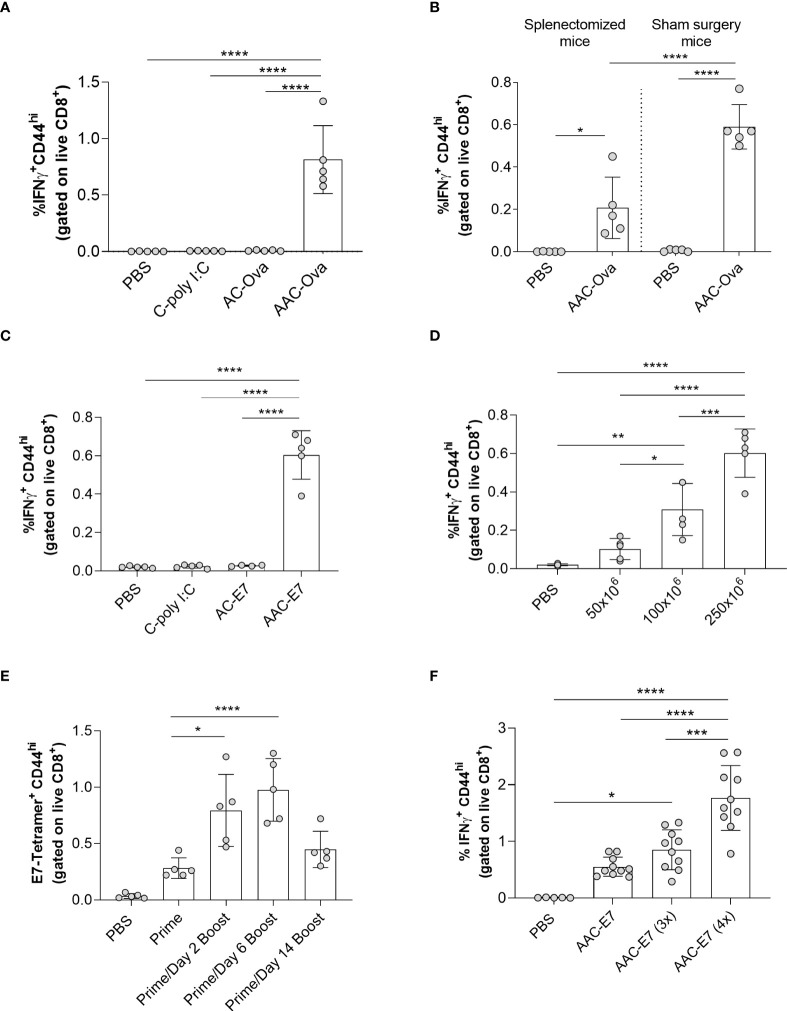
Characterization of AAC-induced CD8^+^ T cell responses *in vivo*. **(A)** Flow analysis of IFNγ^+^ CD44^hi^ of CD8^+^ T cells (referred to as IFNγ^+^ CD8^+^ T cells) from the spleen of mice administered with control vehicle (PBS), C-poly I:C (adjuvant only), AC-Ova (antigen only), or AAC-Ova (antigen and adjuvant). **(B)** CD8^+^ T cell IFNγ responses in the blood following AAC-Ova administration in mice that have undergone splenectomy or sham surgery. **(C)** Flow analysis of IFNγ^+^ CD8^+^ T cells from the spleen of mice administered with control vehicle (PBS), C-poly I:C (adjuvant only), AC-E7 (antigen only), or AAC-E7 (antigen and adjuvant). **(D)** Flow analysis of IFNγ^+^ CD8^+^ T cells from the spleen of mice administered with different doses of AAC-E7. **(E)** Frequency of E7-tetramer^+^ CD8^+^ T cells from the blood for different immunization schedules (250x10^6^ AAC-E7 per animal). **(F)** Flow analysis of IFNγ^+^ CD8^+^ T cells from spleen following dose response to 1, 3 or 4 AAC-E7 immunizations (250x10^6^ AAC-E7 per animal). Figures show one dot per mouse for all studies. *P < 0.05, **P = 0.001, ***P < 0.005, ****P < 0.0001, one-way ANOVA.

The engineered RBCs can be loaded with fluorescently tagged antigen. However, for the carriers to induce antigen-specific immune activation and not tolerance, the addition of both antigen and adjuvant is required (35–38). TLR agonists are used as vaccine adjuvants in anti-cancer therapies because of their ability to activate immune cells and promote inflammation (14, 39–43). To demonstrate that our carriers can activate endogenous T cells as a consequence of the encapsulated antigen and adjuvant, RBCs were processed with the model antigen ovalbumin and poly I:C, a TLR3 agonist, which has been shown to promote cross-presentation by DCs as well as upregulate co-stimulatory and cytokine signals (44–47). RBCs processed with both antigen and adjuvant are herein termed Activating Antigen Carriers (AACs). To distinguish AACs loaded with different antigens, we will use the antigen name following “AAC” (e.g., AAC-Ova). When AAC-Ova clearance kinetics were examined, they showed similar clearance to EC, indicating that the mechanism is largely driven by the cell processing rather the presence of cargo (**Figure S1C**, half-life of 14.9 ± 2.5 minutes). When mice were administered with AAC-Ova and splenocytes examined 7 days post immunization, endogenous Ova-specific CD8^+^ T cell responses were observed (**Figure 2A**) as measured by intracellular cytokine staining (ICS) for IFNγ^+^ CD8^+^ T cells (0.8% ± 0.3%). Indeed, both antigen and adjuvant are required to induce robust IFNγ^+^ CD8^+^ T cell responses as no responses were measured in mice immunized with carriers processed with adjuvant alone (C-poly I:C, 0.01% ± 0.002%) or ovalbumin alone (AC-Ova, 0.01% ± 0.005%) as shown in **Figure 2A**. This potentially indicates that the adjuvanting effects of poly I:C are a local phenomenon that must be delivered in tandem with the antigen of interest as opposed to a systemic phenomenon that can result in non-specific activation. To test whether AACs could activate both CD8^+^ and CD4^+^ T cells, we adoptively transferred (AT) carboxyfluorescein succinimidyl ester (CFSE)-labeled OT-I and OT-II cells into mice with the CD45.1 background. One day later, the mice were immunized with AAC-Ova or phosphate-buffered saline (PBS) control (**Figure S2A**). Proliferation of CD8^+^ and CD4^+^ T cells was observed only in mice immunized with AAC-Ova and not in control mice, as measured by the CFSE profile 3 days post immunization. As the spleen is a known clearance site for aged RBCs and as our carriers exhibit some characteristics similar to those of aged RBCs, the importance of the spleen in mediating this AAC-induced T cell activation was explored.

The spleen is a key organ involved in immune cell activation and clearance of aged or damaged RBCs (48, 49). When splenectomized or age-matched, sham surgery mice were administered PBS or AAC-Ova and the blood collected seven days later, Ova-specific T cell IFNγ levels (**Figure 2B**) were lower in splenectomized mice (0.2% ± 0.2%) compared to sham surgery controls (0.6% ± 0.1%) but were higher than the splenectomized PBS control group (0.00% ± 0.001%). Similar results were observed for CD8^+^ T cell IL-2 levels (**Figure S2B**). This indicates that while the spleen is the primary site for AAC priming of T cells, other organs can act as secondary sites (23, 48, 49). To confirm that we can elicit responses to other relevant tumor antigens, we processed mouse RBCs with a synthetic long peptide (SLP) containing the minimal epitope for the HPV16 viral oncoprotein E7 (**Supplementary Table S1**) in the presence of poly I:C and assessed CD8^+^ T cell responses 7-days following AAC-E7 administration (15, 50–52). As previously observed with the model antigen ovalbumin (**Figure 2A**), co-delivery of E7 SLP and poly I:C to RBCs induced significant responses (0.6% ± 0.1%) compared to control mice administered with PBS (0.02% ± 0.005%), C-poly I:C (0.02% ± 0.01%), or AC-E7 (0.03% ± 0.004%) both for T cell IFNγ levels (**Figure 2C**) and IL-2 levels (**Figure S2C**). Next, we sought to determine the effect of increasing doses of AAC-E7 on the E7-specific CD8^+^ T cell responses. A 2-fold (from 50x10^6^ to 100x10^6^) to 5-fold (50x10^6^ to 250x10^6^) increase in the number of AAC-E7 administered in a single dose resulted in a significant increase in E7-specific CD8^+^ T cell responses (**Figure 2D** and **Figure S2D**).

While a single administration of AAC-E7 did elicit a robust CD8^+^ T cell response, we sought to test different dosing strategies to further enhance T cell responses. To find a more favorable dosing regimen, mice (n = 5 per group) received a single (prime only) or two AAC-E7 immunizations (prime and boost) of 250x10^6^ per animal. Boosting doses were administered 2, 6, or 14 days after the priming dose (**Figure S2E**), and the frequency of E7-specific CD8^+^ T cells in the blood was measured by MHC class I tetramer 7-8 days following the last immunization. The frequency of E7-specific CD8^+^ T cells was significantly higher in the blood of animals which received a booster dose on day 2 (0.4% ± 0.2%) or 6 (1.0% ± 0.3%) compared to prime alone (**Figure 2E**, 0.09% ± 0.05%). While not significantly higher, a response was observed for the booster dose administered on day 14 (0.1% ± 0.06%). To test the effects of multiple boosts, mice were immunized 1-4 times, 7 days apart (**Figure S2F**). Repeated dosing of AAC-E7 significantly increased CD8^+^ T cell responses compared to prime alone (**Figure 2F**, n = 10 mice per AAC-E7 group, n = 5 mice per PBS group).

### AAC *in vivo* uptake induces upregulation of maturation markers of endogenous splenic APCs

**Figure 3:**
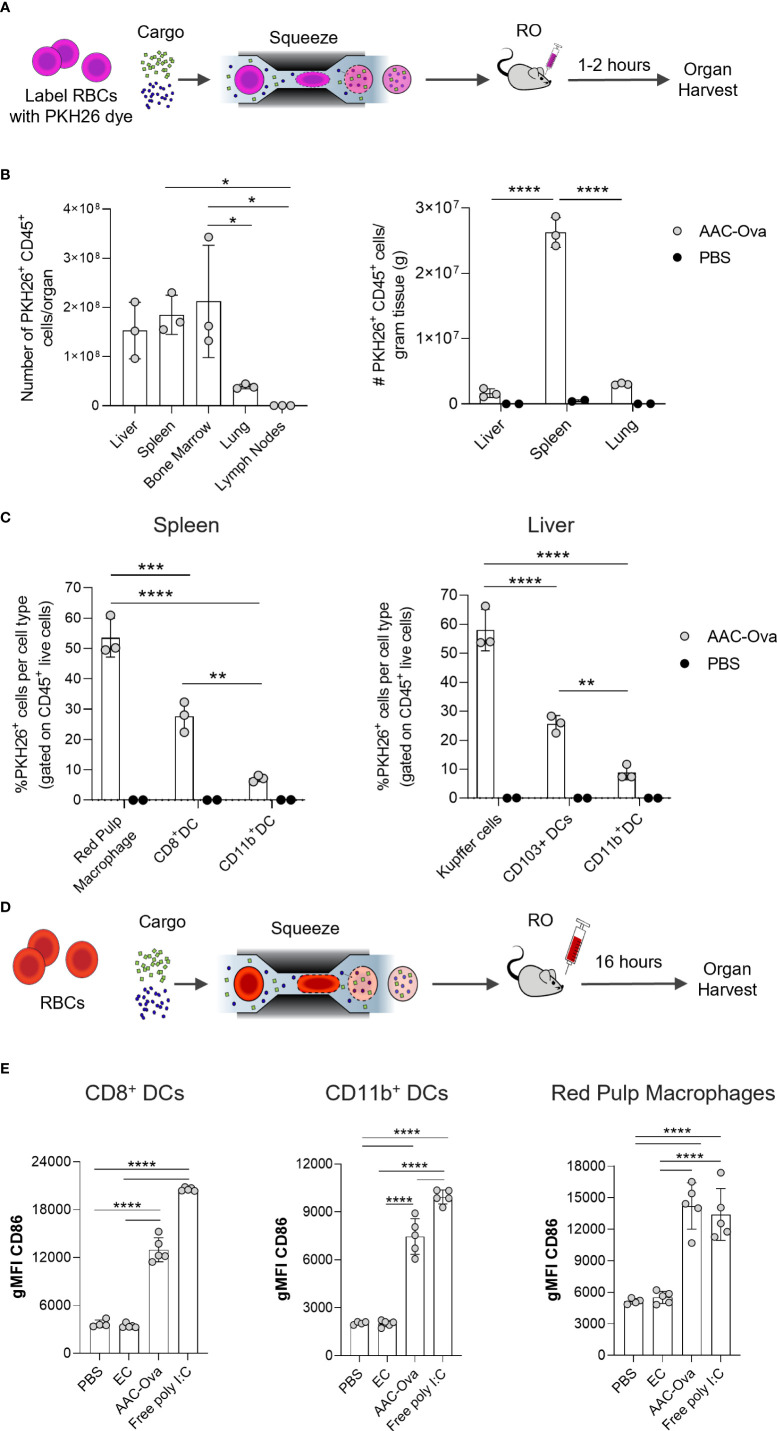
Mouse AACs are rapidly internalized by APCs, inducing maturation *in vivo*. Murine RBCs were squeezed in the presence of Ovalbumin (Ova) and poly I:C to generate AAC-Ova and injected RO at 1x10^9^ per animal. AAC uptake studies **(A–C)** used PKH26-labeled RBCs and organ analysis was performed 1–2 hours after PKH26-labeled AAC-Ova injection. **(B)** The number of PKH26^+^ CD45^+^ cells was determined for each organ. Liver, spleen, and lung were weighed to determine PKH26^+^ CD45^+^ cells per gram tissue for animals injected with AAC-Ova (n = 3 mice) or PBS (n = 2 mice). **(C)** The cell type for PKH26-AAC-Ova uptake was determined in the spleen and liver. For APC maturation studies **(D, E)**, unlabeled RBCs were used for squeeze and organs analyzed the day following AAC-Ova administration. **(E)** Upregulation of CD86 maturation marker on recipient mouse splenic APCs following uptake of AAC-Ova. Figures show one dot per mouse for all studies. n = 2 independent uptake studies, and n = 3 independent maturation studies. *P < 0.05, **P < 0.005, ***P ≤ 0.0005, ****P < 0.0001, one-way ANOVA.

To better understand the priming of AAC-induced T cell responses, the distribution of endogenous AAC uptake was examined. As mentioned previously, aged RBCs are cleared from circulation by phagocytic immune cells in organs of the RES, predominantly liver, spleen, bone marrow, and lung (20, 22, 23). To determine the distribution of carrier uptake in the body, mouse engineered RBCs loaded with ovalbumin and poly I:C were labeled with PKH26 and administered retro-orbitally (RO) into animals (**Figure 3A**). Lymphoid organs were collected and processed 1-2 hours post injection of AAC-Ova and resulting leukocytes examined for PKH26^+^ events. Flow analysis revealed PKH26^+^ leukocytes in the liver, spleen, bone marrow, and to a lesser extent, the lung (**Figure 3B**). As expected, significantly lower PKH26 signal was detected in cells in the lymph nodes, which are not a part of the RES. While there were similar numbers of PKH26^+^ leukocytes in the liver and spleen, the density of leukocyte positive cells found per gram of tissue in the spleen was 15-fold higher than in the liver (**Figure 3B**, right). To further elucidate the distribution of carrier uptake, the type of PKH26^+^ leukocyte was delineated.

As APCs in the liver and spleen are particularly associated with the removal of aged and senescent RBCs, these cells were examined for AAC-Ova uptake (20, 22, 23). In particular, red pulp macrophages (RPM) and DCs in the spleen, and Kupffer cells and DCs in the liver were examined (**Figures S3A, B**) (23, 27, 53). Surface staining in the spleen and liver for these cell types showed that 53.5% ± 6.4% of RPM and 58.0% ± 7.2% of Kupffer cells were PKH26^+^ (**Figure 3C**). Moreover, 27.6% ± 5.0% of CD8^+^ DCs in the spleen and 25.6% ± 2.9% of CD103^+^ DCs in the liver were positive for PKH26. Both types of dendritic cells are reported to be efficient in cross-presentation of cell and non-cell derived antigens (54–57). To a lesser extent, PKH26^+^ AACs were taken up by myeloid CD11b^+^ DCs in both organs (spleen 7.1% ± 1.0%, liver 8.8% ± 2.5%).

We have shown that both antigen and adjuvant co-delivered to AACs are necessary to promote endogenous CD8^+^ T cell responses (**Figure 2A**, **2C**). The internalized adjuvant poly I:C stimulates APCs through TLR3 expressed on the endosomal membrane of macrophages and XCR1^+^ DCs (44–47, 55, 58–60). To demonstrate the activity of delivered poly I:C on APC maturation, spleens of mice immunized with AAC-Ova or EC were harvested 16 hours post administration (**Figure 3D**) and assessed for upregulation of maturation/activation markers (CD86, CD80, CD83, CD40, and MHC class II) on endogenous APCs (**Figure S3C**) (44, 45, 53, 61–64). To confirm that differences observed between carriers with (AAC-Ova) and without (EC) poly I:C are biologically relevant, we compared the maturation marker levels in these groups against mice administered with PBS or free poly I:C (50 µg/animal). While the co-stimulatory molecule CD86 is constitutively expressed on the surface of APCs, its upregulation was observed on CD8^+^ DCs, reported to be the most efficient cross-presenting cells (44–46), on RPMs, and on CD11b^+^ DCs in groups administered with AAC-Ova or free poly I:C relative to control, PBS or EC treated animals (**Figure 3E**). Significant upregulation of other maturation (CD80) and activation markers (CD40, CD83) and MHC class II were detected following administration of AAC-Ova or free poly I:C (**Figure S3D**). As demonstrated above, AAC-enclosed poly I:C was indispensable for the induction of antigen-specific endogenous responses, and its administration resulted in upregulation of maturation markers on endogenous APCs. To assess the safety of poly I:C, a repeat dose study (up to 5 doses, similar to **Figure 2F**) of intravenously administered AAC-E7 was conducted in mice at multiples of the anticipated human dose. Evaluations including histopathology, clinical chemistries, and blood hematology were assessed at the end of the study. Details of the study design and methodologies are provided in **Supplementary Table S2**. The no-observed-adverse-effect level (NOAEL) of mouse AAC-E7 intravenously administered was determined to be approximately 330 μg poly I:C/kg/dose.*in vitro*

### Internalization of human AACs *in vitro* promotes APC maturation and antigen-specific IFNγ responses by CD8^+^ T cells

**Figure 4:**
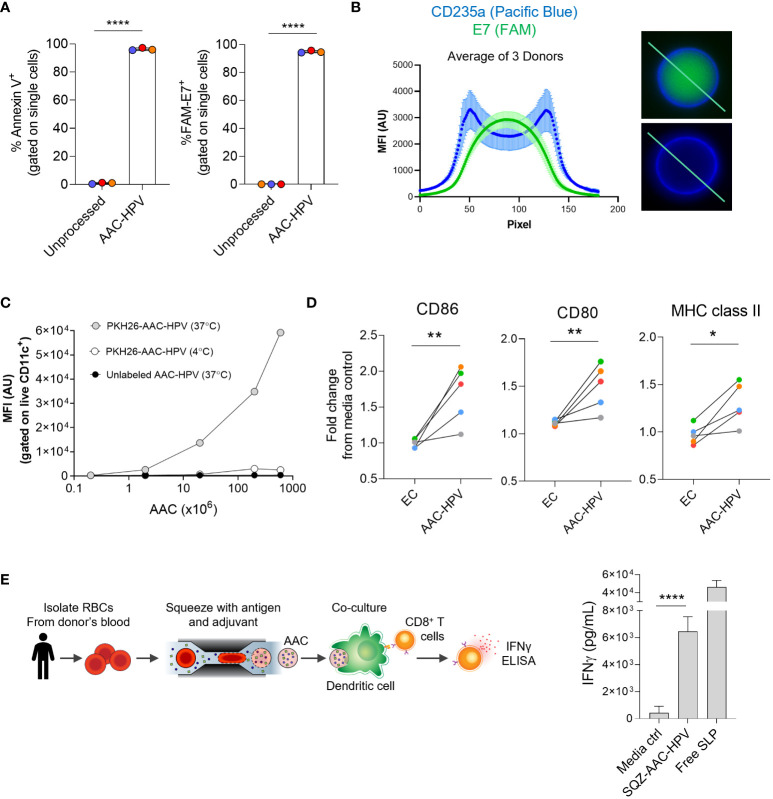
Human AACs show antigen encapsulation and, after uptake, induce MoDC maturation to activate antigen-specific CD8^+^ T cells *in vitro.* **(A)** Annexin V staining and FAM-labeled E7 SLP delivery to human RBCs following squeeze. **(B)** Left: graph displaying mean of 3 donors (see methods section), anti-human CD235a (blue) and FAM-E7 (green) fluorescence intensity along line-scan drawn across the length of the AAC-HPV. Right: line-scan is shown in representative microscopy images of a single human AAC-HPV squeezed with (top) FAM-E7 or (bottom) unlabeled E7 stained with erythrocyte marker anti-human CD235a. **(C)** Uptake of PKH26-AAC-HPV by HLA-A*02^+^ CD11c^+^ MoDCs at 37°C or 4°C. For display purposes, conditions with unlabeled AAC-HPV were plotted on the x-axis at 0.2, since zero cannot be plotted on a log scale (n = 3 independent experiments with 3 distinct RBC donors). **(D)** Expression of maturation markers CD86, CD80 and MHC class II on MoDCs following 2-day culture with AAC-HPV. Data is shown as fold change in gMFI in comparison to media control (n = 5 MoDC donors). Each colored dot represents a different donor. **(E)** Manufacturing scale SQZ-AAC-HPV and HLA-A*02+ MoDCs were cultured overnight with E7_11-20_ -specific CD8^+^ T cells. Supernatants analyzed for IFNγ release by ELISA (n = 6 different RBC donors). *P < 0.05, **P < 0.01, ****P < 0.0001, unpaired t-test.

In order to develop an immunotherapy to treat HPV16^+^ cancer patients, we sought to generate and characterize AACs carrying HPV antigens using human RBCs. Both HPV16 E6 and E7 SLPs (**Supplementary Table S1**) that could target two known HLA-A*02 restricted epitopes (65, 66) were co-squeezed with poly I:C into human RBCs (referred to as AAC-HPV). Similar to the engineered mouse RBCs, engineered human RBCs showed high surface PS presence (annexin V^+^, **Figure 4A** left) compared to unprocessed human RBCs. To examine delivery of the SLPs, AAC-HPV was prepared with a mixture of E6 SLP, poly I:C, and a 5-carboxy-fluorescein (FAM) labeled version of E7 SLP. By flow cytometry analysis, greater than 95% of AAC-HPV were positive for FAM-E7 (**Figure 4A** right, **Figure S4A**, n = 3 different human donors). To confirm that the tumor antigens are encapsulated inside the AAC, RBCs from three donors were loaded with FAM-E7 or unlabeled E7 SLP in the presence of E6 SLP and poly I:C. Samples were stained with human erythrocyte marker anti-CD235a (Pacific Blue) to label the membrane and distinguish intra- versus extracellular regions. Shown in **Figure 4B** are representative images of one donor’s AAC-HPV: FAM-E7 (top) or unlabeled E7 (bottom). Widefield images across the three tested donors are shown in **Figure S4C**. To determine localization of FAM-E7 relative to the cell membrane, FAM and Pacific Blue intensities were plotted using line-scans for each cell and averages from three human donors were compiled (see Methods and **Figure S4D**). The intensity of FAM signal is greatest in the center of the cell and declines towards the edges of the cell (high Pacific Blue intensity regions), suggesting the intracellular localization of squeezed E7 SLP (**Figure 4B** left and **Figure S4B** unlabeled E7 control).

To confirm that the AAC-HPV generated from human donors can be taken up by human APCs *in vitro*, PKH26-labeled engineered RBCs were loaded with poly I:C and E6, E7 SLPs and cultured overnight with human monocyte-derived dendritic cells (MoDCs). To control for AAC-HPV uptake, cells were cultured at either 37°C or 4°C (63). MoDCs cultured at 37°C with PKH26-labeled AAC-HPV showed an increase in PKH26 fluorescence in a dose dependent manner (**Figure 4C**). As expected, cultures at 4°C or those with unlabeled AAC-HPV showed noticeably lower PKH26 fluorescence, suggesting the mechanism of action was related to active phagocytic uptake (63).

We next wanted to examine the activity of co-delivered adjuvant to human APC maturation *via* an *in vitro* system. We incubated MoDCs (n = 3 donors), known to express TLR3 (44, 47), with different concentrations of exogenously added poly I:C and showed upregulation of multiple maturation and activation markers (CD80, CD86, CD83) and MHC class II compared to MoDCs cultured in media alone (**Figure S4E**). Furthermore, MoDCs cultured with AAC-HPV for 46-48 hours showed significant upregulation of CD86, CD80 and MHC class II compared to the MoDCs cultured with EC (both normalized to media controls) using MoDCs from 5 different donors, demonstrating the activity of RBC squeeze delivered poly I:C on human APC maturation (**Figure 4D**).

To demonstrate AAC-HPV elicited antigen-specific CD8^+^ T cell responses, we cultured MoDCs from 6 unique HLA-A*02^+^ donors with AAC-HPV and E7_11-20_ specific CD8^+^ T cells overnight (**Figure 4E**). In these experiments, the AAC-HPV were engineered on a manufacturing scale and cryopreserved (here termed SQZ-AAC-HPV). Following overnight cultures of MoDCs, SQZ-AAC-HPV cells and E7_11-20_ specific CD8^+^ T cells, IFNγ secretion was assessed by ELISA. Significantly higher IFNγ production was measured in SQZ-AAC-HPV groups compared to media control (**Figure 4E**). AAC-induced antigen-specific responses were also confirmed in a cytomegalovirus (CMV) system (**Figure S4F**), suggesting the applicability of the microfluidic platform in different antigen systems in human cells.

In all, we demonstrate, in both mouse and human systems, that encapsulation of antigen and adjuvant into engineered RBCs elicits direct uptake by APCs, followed by APC maturation, and subsequent activation of antigen-specific CD8^+^ T cells. To confirm the therapeutic potential of the AAC platform as an anti-cancer therapy, we performed therapeutic tumor model mouse studies.

### AAC therapeutic treatment slows TC-1 tumor growth and increases infiltration of E7-specific CD8^+^ T cells

**Figure 5:**
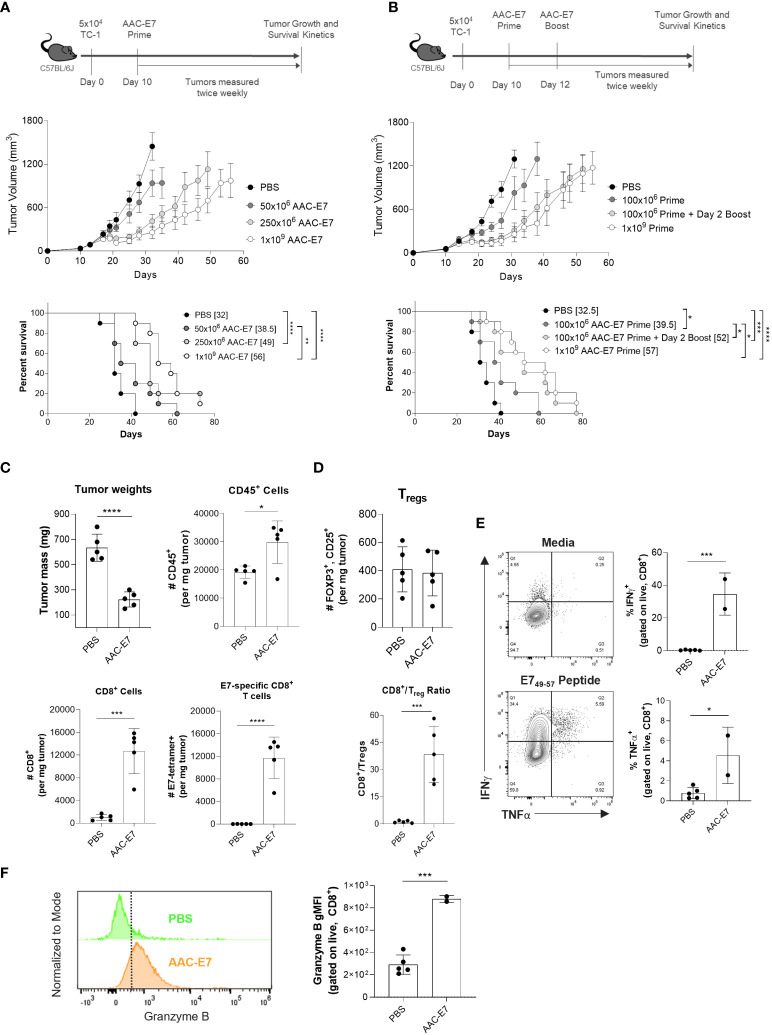
AAC therapeutic treatment primes anti-tumor activity and enhances infiltration of antigen-specific CD8+ T cells in vivo. **(A)** Scheme and results of AAC-E7 single dose response in TC-1 bearing C57BL/6J mice: (top) tumor growth and (bottom) median survival (number in brackets), n = 10 mice per group. **(B)** Scheme and results of AAC-E7 prime/boost on (top) tumor growth and (bottom) survival, n = 10 mice per group. **(C)** Tumor weight in AAC-E7 and PBS-treated groups collected day 23 post TC-1 cell implant. Analysis of tumor infiltrating lymphocytes: total number of CD45^+^, CD8^+^ and tetramer+ E7-specific CD8^+^ T cells per mg tumor, n = 5 mice per group. **(D)** Top: total number Tregs (FOXP3^+^, CD25^+^) per mg tumor. Bottom: ratio of CD8^+^ cells to Tregs in tumors. **(E)** Polyfunctionality (IFNg^+^, TNFα^+^)of tumor infiltrating CD8^+^ T cells upon restimulation (n = 2 for AAC-E7 or n = 5 mice per PBS group). **(F)** Granzyme B^+^ levels in tumor infiltrating CD8^+^ T cells. *P < 0.05, **P < 0.01, ***P < 0.001, ****P < 0.0001, Mantel-Cox test for median survival, other figures analyzed by unpaired t-test.

To evaluate the therapeutic effect of AAC-E7 therapy in the TC-1 tumor model, known to be checkpoint therapy resistant, we first administered a single dose of increasing numbers of AAC-E7 (50x10^6^, 250x10^6^, 1x10^9^), with poly I:C squeezed at 1 mg/mL and monitored the growth of TC-1 tumors (schematic in **Figure 5A**). A single intravenously administered AAC-E7 dose of 250x10^6^ or 1x10^9^ significantly inhibited tumor growth in comparison to PBS-treated mice (**Figure 5A**) while a lower AAC-E7 dose of 50x10^6^ failed to significantly inhibit tumor growth (individual mice shown in **Figure S5A**). In addition, AAC-E7 treatment of TC-1 bearing mice significantly extended median survival in groups treated with 250x10^6^ (49 days) and 1x10^9^ (56 days) AAC-E7 over PBS-treated mice (32 days) (**Figure 5A**). Similar to observations in **Figure 2C**, C-poly I:C treatment showed no benefit in either slowing tumor growth or extending survival relative to PBS-treated mice (**Figure S5B**). These data support the necessity of HPV16 antigen presence for efficacy in the therapeutic AAC-E7 TC-1 model. In a prophylactic setting, a single administration of 1x10^9^ AAC-E7 protected tumor formation in 5 out of 10 animals and extended median survival in AAC-E7 treated animals relative to control mice (**Figure S5D**).

Given the effect of different boosting schedules on the frequency of E7-specific CD8^+^ T cells (**Figure 2E**) and multiple immunizations with AAC-E7 on IFNγ responses (**Figure 2F**), we sought to determine whether additional administrations of AAC-E7 at doses lower than 250x10^6^ would enhance the therapeutic efficacy of AAC-E7 in the TC-1 model. Mice were administered with a single dose of 100x10^6^ AAC-E7 on day 10, or two 100x10^6^ doses administered on day 10 and 12 post TC-1 implant (**Figure 5B**). To separate the effect of boosting from the effect of increasing the overall dose, an additional group of mice were administered with a single dose of 1x10^9^ AAC-E7 on day 10 post TC-1 implant. Two administrations of AAC-E7 at 100x10^6^ were more efficacious in slowing tumor growth than a single administration of 100x10^6^ AAC-E7 and comparable to a single administration of 1x10^9^ AAC-E7 (**Figure 5B**). Treatment with two doses of 100x10^6^ or one dose of 1x10^9^ AAC-E7 significantly extended the median survival compared to a single 100x10^6^ AAC-E7 dose from 39.5 to 52 days (100x10^6^ prime/boost), or 57 days (1x10^9^ prime). Despite a 5-fold difference in the total number of cells used for immunization, the tumor growth kinetics was similar between the 100x106 prime/boost and 1x10^9^ prime groups.

To investigate changes in the tumor microenvironment upon administration of AAC-E7, mice were administered with either PBS, or two doses of 250x10^6^ AAC-E7 on day 14 and 16 post TC-1 implant (n = 5 mice per group). For the analysis of tumor infiltrating lymphocytes (TILs), tumors were allowed to grow larger before the first AAC-E7 dose (day 14 vs. day 10 in study shown in **Figure 5A**) to increase tumor cell recovery for analysis. The average tumor mass in AAC-E7 treated groups was significantly lower than those in PBS-treated animals (**Figure 5C**) when tumors were harvested for TIL analysis (day 23 post TC-1 implant). A significant increase in the total number of CD45^+^ cells per mg tumor mass (**Figure 5C**) as well as percent of CD45^+^ cells (**Figure S5E**) was observed in AAC-E7 treated animals (49.3% ± 9.1%) compared to PBS-treated mice (17.0% ± 4.1%). In AAC-immunized animals, CD8^+^ T cells comprised 41.8% ± 5.5% of all infiltrating lymphocytes (**Figure S5E**). The AAC-E7 group showed 13-fold more CD8^+^ T cells per mg of tumor (**Figure 5C**) than the PBS group (**Figure 5C**). More than 90% of recruited CD8^+^ T cells were E7-specific (**Figure S5E**, 92.4% ± 1.3%) in AAC-E7 treated animals. This amounted to 900-fold more E7-specific CD8^+^ T cells in AAC-E7 treated animals than in PBS-treated, more than 50% of which were proliferating Ki-67^+^ cells (**Figure S5F**). In addition to the high influx of E7-specific CD8^+^ T cells in tumors of AAC-treated animals, a ratio of CD8^+^ T cells over regulatory T cells (Tregs, FOXP3^+^CD25^+^) was significantly elevated (> 30-fold) compared to PBS-treated animals, while the total number of Tregs remained similar, irrespective of treatment (**Figure 5D**).

To assess the functionality of AAC-induced infiltrated CD8^+^ T cells, ICS analysis was performed on TILs. This revealed a significant increase in the frequency of polyfunctional (IFNγ^+^, TNFα^+^) CD8^+^ T cells (**Figure 5E**) and increased Granzyme B production (**Figure 5F**). Increased polyfunctionality of CD8^+^ T cells was observed in the periphery (spleen) of AAC-E7 treated animals as well (**Figure S5G**). Splenocyte analysis also showed an increase in E7-specific CD8^+^ T cell frequency, more than 50% of which were Ki-67^+^ (**Figure S5H**).

**Figure 6:**
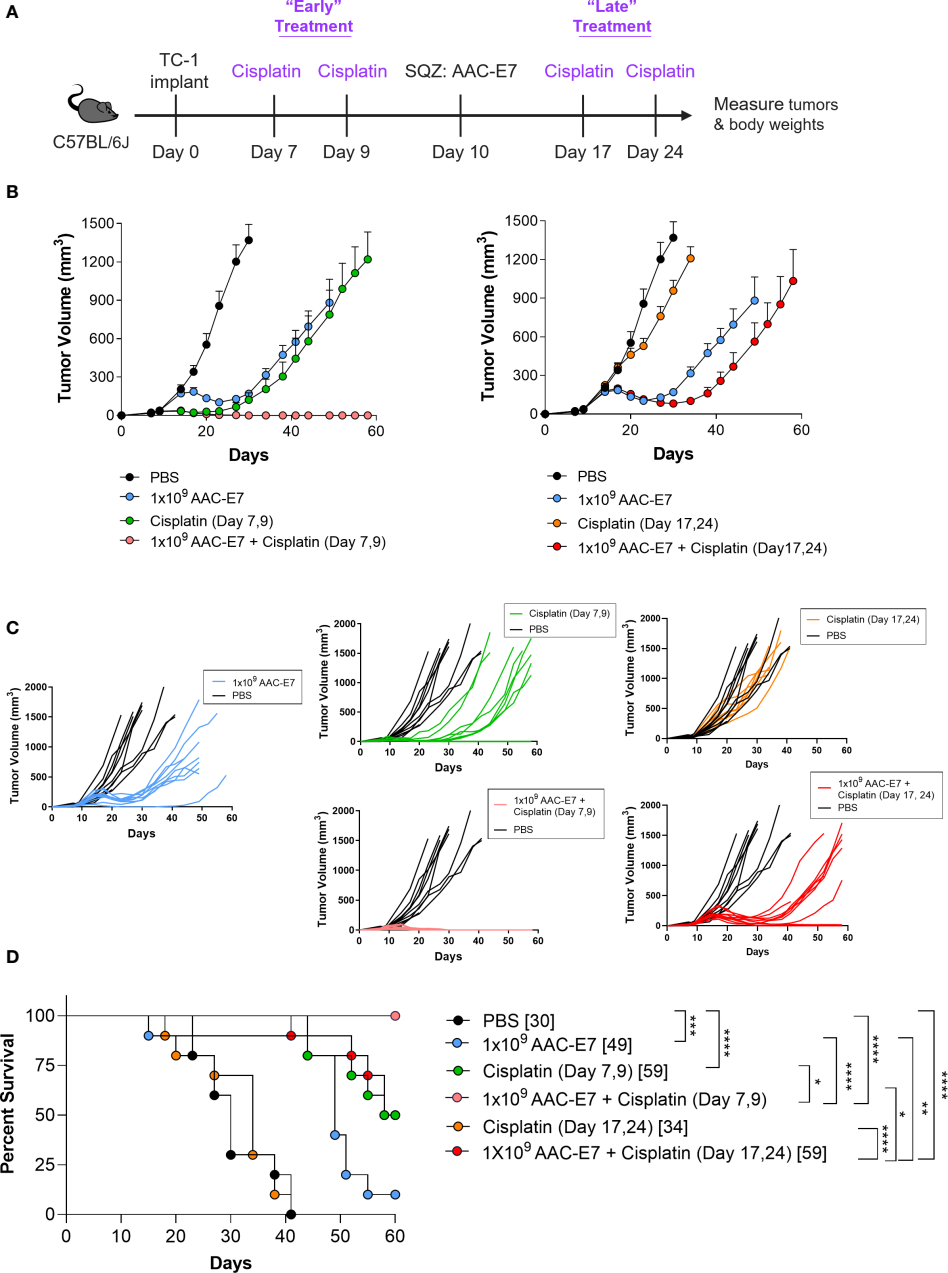
Synergistic therapeutic effect of AACs and chemotherapy combination. **(A)** Schematic of Cisplatin and AAC-E7 dosing. Early (two doses: day 7, 9 post TC-1 implant) and late (two doses: day 17, 24 post TC-1 implant) Cisplatin dosing was administered as monotherapy or in combination with AAC-E7. **(B)** Tumor growth curves for PBS, AAC-E7 and Cisplatin monotherapy, or combination therapy. The figures show the same PBS and AAC-E7 monotherapy treatment groups overlayed with early (left) and late (right) Cisplatin dosing. **(C)** Spider plots of individual mice per each treatment group. **(D)** Median survival (n = 10 mice per group). *P < 0.05, **P < 0.01, ***P < 0.001, ****P < 0.0001, Mantel-Cox test for median survival.

DNA damaging chemotherapeutic agents are a common approach to treating cancer and can be combined with other methods (2, 67). A treatment with two or more therapeutic agents often enhances the therapy’s efficacy by complementing modes of action of monotherapies in a synergistic fashion. Here, we wanted to investigate whether combining the AAC-E7 immunization with the DNA damaging agent Cisplatin, could further enhance the therapeutic efficacy in the TC-1 tumor model. Cisplatin is an approved chemotherapeutic agent for the first line treatment of ovarian, head and neck, bladder, and other types of cancers (67, 68). To explore different combination scenarios with Cisplatin, mice were administered (via intraperitoneal injection) with two low doses (5 mg/kg) of Cisplatin either before AAC-E7 (“early”, day 7 and 9 post TC-1 implant) or after AAC-E7 (“late”, day 17 and 24 post TC-1 implant) immunization (**Figure 6A**). While the early Cisplatin and AAC-E7 monotherapies were individually effective in slowing down TC-1 tumor growth relative to PBS-treated control mice (**Figure 6B** left, **6C**), the combination of these therapies completely cleared tumors in all treated animals: 10/10 animals remained tumor-free on day 72 post TC-1 implant (data shown up to day 60). A combination of AAC-E7 with the late Cisplatin treatment was able to slow tumor growth, more so than the monotherapies, but to a lesser extent than the early Cisplatin with AAC-E7 combination group (**Figure 6B** right, **6C**). The late Cisplatin monotherapy was indistinguishable from the PBS-treated mice. Both combination regimens significantly extended the median survival of treated animals compared to AAC-E7 monotherapy or resulted in complete tumor clearance (**Figures 6D**). To assess memory formation in tumor-free mice post primary TC-1 challenge, tumor-free mice in the “early” Cisplatin treatment combination group were rechallenged on day 73 post TC-1 implant. At the end of the study, 60 days following the secondary challenge, 4 mice were still tumor-free, tumor growth was visibly slowed in 4 animals, and tumor volumes in 2 animals were indistinguishable from control mice (**Figures S6A, B**). Together, these results demonstrate a potential for a successful, memory inducing, therapeutic combination of AAC therapy with an approved anti-cancer agent.

## Discussion

Here, we demonstrate our RBC-derived AAC platform targets encapsulated cancer antigens and adjuvant to endogenous APCs, which in turn elicit robust anti-tumor CD8^+^ T cell responses *via* cross-presentation. Our approach generates engineered cells easily loaded with a variety of materials that exhibit FSC^low^, SSC^low^ with PS exposed on the outer leaflet of the membrane as compared to unprocessed (non-squeezed) RBCs. The observed phenotype closely matches aged and senescent RBCs (18, 20, 27) and utilizes the natural process of eryptosis (RBC cell death) to directly mark these engineered cells for uptake by professional APCs at T cell priming sites. As shown, AACs are cleared from circulation within the first hour of administration, compared to unprocessed RBCs which persist over the course of multiple days. While it is possible that other scavenger receptors could be involved in the internalization of carriers, we believe the reproducibly high PS exposure on the surface of the engineered carriers suggests this as the MOA for the uptake by APCs.

When labeled AACs were tracked *in vivo*, they were primarily taken up in organs of the RES, in particular the spleen and the liver. Macrophages and dendritic cells made up the majority of cell subsets responsible for the uptake. Yet, because the *in vivo* CD8^+^ T cell responses were dependent not only on the presence of antigen but also on the presence of TLR3 agonist poly I:C, it is likely that the TLR3 sensitive APCs are the key cell types in the AAC MOA. Furthermore, the reduction in antigen-specific CD8^+^ T cell responses in mice subjected to splenectomy suggests that splenic APCs play a significant role in processing AAC antigens for presentation to CD8^+^ T cells. Among splenic APCs, CD8^+^ DCs are known to efficiently cross-present antigens, apoptotic cell-associated antigens in particular (55, 69), and likely are a key player in the AAC-induced CD8^+^ T cell responses. However, macrophages are reported to be TLR3 positive and capable of cross-presentation as well (60), and therefore may also contribute to AAC-induced CD8^+^ T cell responses. While RPM have typically been associated with clearance of dying or damaged RBCs as well as parasitic, bacterial or fungal infections (70), work from the Garbi and Kurts groups (71) showed the importance of RPMs in promoting CD8^+^ T cell responses. Using Spi-C knockout mice, which lack RPMs, they demonstrated that RPMs play a significant role in early (< 3 hours post antigen exposure) protein processing and cross-presentation to elicit effector cytotoxic CD8^+^ T cell responses, while classical type 1 dendritic cells (cDC1s) appear to take over that role 3 hours post antigen exposure and are more important for inducing memory T cell responses. How this apparent “handoff” of CD8^+^ T cell priming works in the AAC MOA will be interesting to explore in future studies. Our results also demonstrate that AAC therapy can induce CD4^+^ T activation (**Figure S2**). This is likely linked to the ability of AACs to induce MHC class II upregulation on APCs both in mice (**Figure S3D**) and humans cells (**Figure 4D**). Future studies could examine which subset(s) of CD4^+^ T cells are expanding and whether they display a cytotoxic signature (SLAMF7, perforin, granzyme, Fas ligand expression). These cells, as recently detailed by Cachot et al. (72), have been identified in multiple cancer types and may be linked to better patient outcomes.

While AACs alone were able to expand polyfunctional tumor-specific CD8^+^ T cells that infiltrated tumors and slowed tumor growth, the tumors eventually relapsed in the therapeutic setting. As with most immune modulating therapies, it is important to consider potential combination therapies that may have synergistic activity. We investigated the use of the chemotherapeutic agent Cisplatin, which was able to cause complete tumor regression when combined with AACs, when neither therapy alone was sufficient to cause complete regression. We speculate that this synergistic activity is due to the combined pressures of DNA damage and subsequent inflammation from Cisplatin and cytotoxicity of E7-specific CD8^+^ T cells from AAC-E7 immunization (2, 73). These combined activities may also lead to exposure of additional tumor-associated antigens, and further promote T cell activation. This is one example of potential combinations, but others should be considered. Given the apparent role of DCs in driving responses, immune stimulators that augment priming, such as anti-CD40 or anti-CTLA4 could be explored (3, 67, 74–77). During expansion of antigen-specific CD8^+^ T cells, delivery of cytokines may augment proliferation of CD8^+^ T cells and enhance their activity (78). Once in the tumor, therapeutic agents that modulate the tumor microenvironment such as anti-PD1, anti-PD-L1, anti-TGFβ, anti-TIM-3, and others could be viable options (77, 79, 80).

To demonstrate that the AAC therapy is suitable for use in a clinical setting, we cryopreserved human AACs delivered with HPV16 E6 and E7 antigens and poly I:C (SQZ-AAC-HPV). This demonstrates that an AAC drug product could be cryopreserved, stored for transportation, and maintain functionality, allowing for one blood draw to generate multiple drug batches and providing flexibility for patient dosing schedules. Indeed, post thaw, human SQZ-AAC-HPV cells were able to induce antigen specific IFNγ responses from E7_11-20_ specific human CD8^+^ T cells cultured with MoDCs.

Reported immunotherapies are still facing challenges that the targeting features of an AAC therapy can potentially address. While the administration of free cancer antigens and adjuvant(s) elicits responses and provides all the necessary signals for T cell activation (2, 24), it lacks localized delivery of immunostimulatory factors to endogenous APCs and instead can promote systemic inflammation and premature clearance of antigen with insufficient T cell priming. AAC therapy, in comparison, enables encapsulated material to be taken up, processed and presented by relevant APCs. Alternative strategies such as*, ex vivo* incubation of patient-derived DCs with antigens and maturation cocktails, can alleviate this issue but result in a heterogenous DC phenotype and require lengthy manufacturing. Some approaches also seek to circumvent the lengthy manufacturing and exposure of activating material by using antigen encoding mRNA complexed with lipid nanoparticles to target endogenous APCs within lymphoid compartments (43). However, such therapies may not ensure highly specific targeting of the APCs and are often focused on the induction of signal 1, with potentially weaker activation of signals 2 and 3, as the APCs processing the antigen may not be simultaneously affected by the lipid adjuvant (43).

Nanocarrier-based cancer therapies are an evolving field that is perhaps the most direct comparator to AACs. While a benefit of nanomedicine is, as already described for AACs, the encapsulation of drug material to prevent drug degradation and the goal is to provide more targeted therapy, progress is still needed to shuttle these particles towards the relevant APCs for uptake and activation (43, 81, 82). Within the nanoparticle community, work is still underway to determine which formulation of biocompatible materials generates the optimal response without off-target effects (6, 7). In contrast, the physiological nature of AACs ensures target delivery and rapid clearance by APCs while keeping the manufacturing process rapid and using the patient's own RBCs instead of synthetic lipids. While many groups are working to conquer these limitations (7, 43, 83, 84), the best example being the success of the COVID mRNA vaccines (85), work is still needed to obtain a successful nanoparticle-based cancer therapy that can generate robust CD8^+^T cell responses (83). Viral vectors too could be compared against AACs. However, adenovirus-based vaccines may be cleared by the patient’s pre-existing neutralizing antibodies or another immunological response (84), and can carry the risk of insertional mutagenesis (86) with potentially severe immunological responses leading to adverse events (i.e., cytokine storm) (84). Other emerging RBC-based cancer therapies may be able to address some of the aforementioned challenges however they often involve lengthy and complex manufacturing. They can be reliant on prolonged dialysis, binding materials to the outer membrane of RBCs, or genetic manipulation of cells (11–14, 36). In comparison, the Cell Squeeze^®^ process applies a transient and quick mechanical disruption in a microfluidic passage to encapsulate both adjuvant and antigen into RBCs with rapid and scalable manufacturing (< 24 hours; vein-to-vein < 10 days). Once administered, the AACs promote rapid, targeted, and simultaneous delivery of antigens and adjuvant to endogenous APCs. The AAC platform has the potential to be an efficacious standalone therapy and a desirable partner in different combinatorial approaches to address unmet needs in the field of cancer immunotherapy.

In summary, by engineering RBCs to encapsulate tumor antigen(s) and adjuvant in concert with the exposure of phosphatidylserine, we can harness the natural process of eryptosis-mediated phagocytosis by professional APCs to drive antigen presentation and T cell activation, as demonstrated with *in vivo* mouse and *in vitro* human models. This approach to generate RBC therapeutics can be easily tailored to deliver a plethora of antigen and adjuvant materials, and other possible agents, to enhance different aspects of the immunity cycle, and supports the further study and clinical implementation of AACs as a cancer immunotherapy platform.

## Materials and methods

### Mice

All studies were carried out according to protocols established by the American Association for Laboratory Animal Science (IACUC) committee at SQZ Biotechnologies Company. All animals had specific pathogen free (SPF) status when acquired and were maintained in an SPF facility. C57BL/6J mice were purchased from The Jackson Laboratory (Bar Harbor, ME, USA). Female donor mice used for blood squeezes were between 9-22 weeks old while recipient mice were 9-12 weeks. Splenectomy and sham surgeries were performed by The Jackson Laboratory at 7 weeks of age prior to delivery of animals.

### Cell processing

RBCs were isolated from mouse or human whole blood by Ficoll gradient (GE Healthcare, Chicago, IL, USA) and squeezed at 1x10^9^ cells/mL, 50-70 pound per square inch (PSI) for mouse and at 2x10^9^ cells/mL, 60 PSI for human in either PBS or RPMI 1640 (Gibco, Waltham, MA, USA) delivery buffer using a custom-made microfluidic system (HT-10-022-70, Silex, Boston, MA, USA). Blood was pooled from multiple, syngeneic donor mice to generate RBC material for the studies mentioned below. Mouse carriers were generated by squeezing RBCs with Ovalbumin-Alexa Fluor 647 (200 µg/mL, Thermo Fisher Scientific, Pittsburgh, PA, USA), or 200 µg/mL Endofit Ovalbumin (InVivoGen, San Diego, CA, USA), or 100 μM mouse-E7 SLP (Biosyntan GmbH, Berlin, Germany), RPMI 1640 (Gibco), and/or 1 mg/mL low molecular weight poly I:C (Dalton Pharma Services, Toronto, Ontario).

Human carriers were generated by squeezing RBCs with E6 SLP (50 µM, Biosyntan GmbH) and human-E7 SLP (200 µM, Biosyntan GmbH) or CMV pp65 SLP (Biosyntan GmbH, 100 µM) and 1 mg/mL low molecular weight poly I:C (Dalton Pharma Services). For data in **Figure S4F**, squeezes were performed without poly I:C, which was instead added exogenously 3 hours after AAC addition, at the indicated concentrations. The cargo solution was mixed with RBCs immediately prior to squeeze. This occurred at room temperature for all mouse squeezes while human HPV16 and CMV SLP squeezes occurred on ice. After a 1-hour post squeeze rest, cells were washed 4-7 times with PBS (Gibco), spinning at 8000xg, 5 min, acceleration = 9, brake = 4, at room temperature before use. SLP sequences are supplied in **Supplementary Table S1**. Cryopreservation was performed in CS2 (BioLife Solutions, Bothell, WA, USA) at a target concentration of 1 x10^9^ cells/mL in AT-10 vials (Aseptic Technologies, Raleigh, NC, USA).

### Multicolor flow cytometry

All flow cytometry was performed on an Attune NxT flow cytometer (Thermo Fisher Scientific) and analyzed by FlowJo-v10 (Ashland, OR, USA).

### *In vitro* characterization of mouse and human carriers

After the post squeeze rest and washes (see above), carriers were stained (1x10^8^/mL) for characterization and analyzed (2x10^6^/mL) by flow cytometry in Annexin V Binding Buffer (Biolegend, San Diego, CA, USA) using the panels described in **Supplementary Table S3**.

### Assessment of PKH26-AAC-Ova clearance and uptake

For clearance studies, mouse RBCs were labeled with cell membrane dye PKH26 (Sigma-Aldrich, St. Louis, MA, USA) according to the manufacturer’s directions. Labeled RBCs were squeezed with Endofit Ovalbumin (InVivoGen) and poly I:C (Dalton Pharma Services) in PBS as described above to generate PKH26-labeled AAC-Ova cells. These were injected (1x10^9^ per mouse) RO into C57BL/6J recipients. Blood samples were collected into citrate phosphate dextrose-adenine 1 (Sigma-Aldrich) 0, 15 min, 30 min, 1 hour, 4 hours, 96 hours post PKH26-AAC-Ova injection and analyzed for PKH26^+^, CD45^+^ events by flow cytometry.

For biodistribution studies, tissues were collected 1-2 hours after PKH26-labeled AAC-Ova administration and weighed. Tissues were minced by razor blades into 1-2 mm pieces, incubated in collagenase XI (Sigma-Aldrich, 0.38% w/v) in Hanks’ Balanced Salt Solution (Gibco) for 30 minutes at 37°C and processed into single cell suspensions. Liver samples were further processed for isolation of non-parenchymal cells by a Percoll gradient (GE Healthcare). Cells were blocked using FcR Blocking Reagent (Miltenyi Biotech, Bergisch Gladback, Germany) according to manufacturer’s instructions and staining performed with the panel described in **Supplementary Table S4**. Cells were fixed in 4% paraformaldehyde (Electron Microscopy Sciences, Hatfield, PA, USA) prior to acquisition. Cell populations were defined (**Figure S3**) as follows after the live, CD45^+^, CD19^-^/NK1.1^-^ gates: splenic red pulp macrophages (CD11b^low/-^, F4/80^+^), splenic CD8^+^ DCs (CD11c^hi^, MHC class II^hi^, CD8^+^), CD11b^+^ DCs (CD11c^hi^, MHC class II^hi^, CD11b^+^), Kuppfer cells (CD11b^low/-^, F4/80^+^), liver CD103^+^ DCs (CD11c^hi^, MHC class II^hi^, CD103^+^).

For endogenous APC maturation studies, mice were administered (RO) PBS, 50 µg/animal free low molecular weight poly I:C (Dalton Pharma Services), 1x10^9^ EC or 1x10^9^ AAC-Ova and spleens were harvested 16 hours later. Tissues were digested in collagenase XI (Sigma-Aldrich) as described above. Single cell suspensions were blocked using FcR Blocking Reagent (Miltenyi Biotech) according to manufacturer’s instructions and stained with the panel described in **Supplementary Table S5**. Cells were fixed in 4% paraformaldehyde (Electron Microscopy Sciences) prior to acquisition. Expression is quantified as geometric mean fluorescence intensity (gMFI).

### Endogenous responses: OVA model and E7 model

Mouse AAC-Ova was generated as stated above by squeezing RBCs with EndoFit Ovalbumin (InVivoGen) and poly I:C (InVivoGen), while AAC-E7 was generated by squeezing mouse RBCs with mouse-E7 SLP (Biosyntan GmbH) and poly I:C (Dalton Pharma Services). Either were injected RO at 250x10^6^ per C57BL/6J recipient (unless otherwise indicated) on day 0. In cases of multiple immunizations, see **Figure S2E** and **S2F** for a timeline. Seven days post last immunization, spleens (or blood in the case of splenectomized mouse studies) were harvested, processed into single cells, and co-cultured with appropriate stimulation, anti-CD28 (8 µg/mL, eBioscience, San Diego, CA, USA) and either Ova_257-264_ (1 µg/mL, SIINFEKL, AnaSpec, Freemont, CA, USA) or E7_49-57_ (4 µg/mL, AnaSpec) with sequences found in **Supplementary Table S1**, for one hour and for an additional four hours in the presence of GolgiStop/GolgiPlug (BD Bioscience, Franklin Lakes, NJ, USA). Following restimulation, cells were stained for flow cytometry analysis using BD Bioscience FACS lysis solution and Fixation/Permeabilization kit according to manufacturer’s instructions and with the panel in **Supplementary Table S6**. For tetramer staining, the panel in **Supplementary Table S7** was used.

### OT-I/OT-II transfer

Female 8 to 10-week-old Jackson mice were used: C57BL/6-Tg (TcraTcrb)1100Mjb/J (i.e. OT-I), C57BL/6-Tg (TcraTcrb)425Cbn/J (i.e. OT-II), and B6.SJL-Ptprca Pepcb/BoyJ (i.e. CD45.1). Cells were isolated using STEMCELL kits (Vancouver, Canada): CD8^+^ T cells from OT-I mice using EasySep Mouse CD8^+^ T cell isolation kit and CD4^+^ T cells from OT-II mice using EasySep Mouse CD4^+^ T cell isolation kit. T cells were labeled with CellTrace CFSE Cell Proliferation Kit (Invitrogen, Carlsbad, CA, USA). CFSE labeled CD8^+^ T cells and CD4^+^ T cells were administered RO (2.5x10^6^ per mouse of each cell type) on day 0 to CD45.1 recipients. Murine RBCs were squeezed as stated above with EndoFit Ovalbumin (InVivoGen) and poly I:C (InVivoGen). These AAC-Ova were injected RO (250x10^6^ per mouse) on day 1 into the same CD45.1 recipients. Some CD45.1 recipients received PBS as a control. After 3 days, lymph nodes (axillary, brachial, cervical, mesenteric, inguinal) and spleens were processed into a single cell solution, and proliferation of OT-I and OT-II cells was assessed by flow cytometry using the panel in **Supplementary Table S8**.

### Microscopy

Human AAC-HPV were generated from 3 separate donors by squeezing RBCs with E6 SLP (50 µM, Biosyntan GmbH), poly I:C (Dalton Pharma Services), and either unlabeled human-E7 SLP or 5-carboxy-fluorescein (FAM)-labeled human-E7 SLP (200 µM, Biosyntan GmbH) as stated above. After squeeze, AAC-HPV were rested for 20 min at 4°C then 1 hour at 37°C and washed seven times. Samples were brought up to 2x10^9^/mL in PBS. Samples were then stained with anti-human CD235a Pacific Blue (BioLegend) for 15 minutes at room temperature. Unstained and single stained samples (squeezed with unlabeled E7) were also prepared. AAC-HPV were seeded (2.9x10^6^ per microscope slide) and mounted with a coverslip (Fisher Scientific, Waltham, MA, USA). Samples were imaged using a Zeiss Axio Imager fluorescence microscope (Jena, Germany), 63x objective, 1.4 numerical aperture, 2752 x 2208 pixels, with a pixel size of 4.5 µm. Therefore, 1 pixel = 0.072 µm. Per donor, 3 – 5 fields of view (FOV) for FAM-E7 and 1-2 FOV for unlabeled E7 squeezes were acquired, each with cells selected at random for imaging. For analysis, 1-2 regions of interest (ROI, each with 9-12 AACs) were selected per FOV (see **Figure S4D** for scheme). Line-scans were generated for each AAC using Fiji image analysis software (Bethesda, MA, USA) to collect FAM and Pacific Blue fluorescence intensities. Background subtraction was performed by determining the minimum fluorescent intensity to calculate arbitrary units (AU) for each fluorophore across all pixels of the line-scan and subtracting this from each pixel’s fluorescence intensity along the line-scan to generate a relative fluorescence intensity. The mean and standard deviation of all relative fluorescence intensities was calculated for all cells in a single ROI to generate one representative line-scan per ROI. The same procedure was applied to all FOV for each given donor. Such generated “mathematical” line-scans were averaged to generate a single summary line-scan for each donor.

### Monocyte derived dendritic cells

Human monocytes were purified from HLA-A*02:01^+^ typed leukopaks (STEMCELL Technologies) using the EasySep monocyte enrichment kit without CD16 depletion (STEMCELL Technologies) according to the manufacturer’s directions. Greater that 85% monocyte purity and HLA-A*02 expression was confirmed by flow cytometry using the panel described in **Supplementary Table S9** before differentiation was initiated. Monocytes were cultured at 37°C in CellGenix GMP DC Medium (CellGenix, Freiburg, Germany), human AB serum (HS, 5%, Sigma-Aldrich), Penicillin-Streptomycin (Pen/Strep, 1%, Corning, Corning, NY, USA), L-glutamine (2 mM, Gibco), rhGMCSF (1000 U/mL) and rhIL-4 (800 U/mL, R&D Systems, Minneapolis, MN, USA) on Nunc EasYFlask T-175 cm^2^ flasks (Thermo Fisher Scientific) before supplementing with fresh cytokines on day 3, then collecting and cryopreserving the cells in CryoStor CS10 (Biolife Solutions, Bothell, WA, USA) on day 4. Differentiation was confirmed by flow cytometry using the panel described in **Supplementary Table S10**. The day before MoDCs were needed, cells were thawed and cultured overnight (6.6 x10^5^/mL) in the same differentiation media.

### Human AAC-HPV uptake assay

MoDCs were seeded (1.5x10^5^/well) on 96-well, flat bottom, ultra-low attachment plates (Corning). For 4°C cultures, MoDCs were incubated at 37°C for 2 hours before moving to 4°C for 6-7 hours. For 37°C cultures, MoDCs were incubated at 37°C for 6-7 hours. Human RBCs were labeled with PKH26 (Sigma-Aldrich) according to the manufacturer’s directions and squeeze processed with E6 and E7 SLP and poly I:C as stated above. Resulting PKH26-labeled AAC-HPV were washed post squeeze before plated at the indicated density with MoDCs (post 6-7 hr incubation), X-VIVO 15 (Lonza, Basel, Switzerland) and HS (5%, Sigma-Aldrich) and cultured at the indicated temperature for 16-18 hours. Cells were then collected and stained for CD11c^+^, PKH26^+^ double positive events by flow cytometry using the panel described in **Supplementary Table S11**.

### Human MoDC maturation assay

MoDCs were seeded (1.5x10^5^/well) on 96-well flat-bottom, ultra-low attachment plates and incubated at 37°C for 1-2 hours before addition of AAC-HPV (6x10^8^/well) or X-VIVO 15 (Lonza). HS (5%) was added before culturing the AACs for 46 hours in a 37°C incubator. For the studies on exogenously added free poly I:C, MoDCs were cultured with low molecular weight poly I:C (Dalton Pharma Services, 10, 50, or 250 µg/mL) for 46 hours. Cells were then collected and stained for maturation markers by flow cytometry using the panel described in **Supplementary Table S12**.

### Human MoDC, SQZ-AAC-HPV and AAC-CMV co-culture assay

Human AAC-HPV were generated by squeezing unlabeled human-E7 and E6 (Biosyntan GmbH) SLPs and poly I:C (Dalton Pharma Services) as described above. AAC-CMV were generated by squeezing unlabeled CMV pp65 SLP without poly I:C, which was instead added exogenously to co-cultures with MoDCs and antigen-specific CD8^+^ T cells (**Figure S4F**). SQZ-AAC-HPV (**Figure 4**) were generated on a manufacturing scale while AAC-CMV (**Figure S4F**) was performed on the research scale. MoDCs were seeded (1.5x10^5^/well) on 96-well flat bottom, ultra-low attachment plates (Corning) and incubated at 37°C for 2-3 hrs before addition of SQZ-AAC-HPV (6x10^8^/well), or AAC-CMV (300x10^6^/well), human-E7 SLP or CMV or pp65 SLP (2 µM, Biosyntan GmbH), or X-VIVO 15 (Lonza). After 20 min, lipopolysaccharide (LPS, 20 µg/mL, *In vivo*Gen), HS (5%, Sigma-Aldrich) and E7_11-20_ or pp65_495-503_ CD8^+^ T cell-responders (5x10^4^/well, Cellero, Lowell, MA, USA) were added. After 24 hrs, plated cells were spun at 2000xg, 10 min, room temperature before supernatants were collected for analysis by human IFNγ ELISA (BioLegend).

### TC-1 tumor model

TC-1 cells were obtained from Dr. T.C. Wu (Johns Hopkins) and cultured in TC-1 growth media: Gibco 1 mM sodium pyruvate, 1x non-essential amino acids, 400 µg/mL Geneticin selective antibiotic (G418 Sulfate), RPMI 1640, 10% fetal bovine serum and 1% Pen/Strep. TC-1 cells were thawed and cultured at a density of 3x10^4^ cells/cm^2^ for the first 48 hours and then split to a density of 3x10^4^ cells/cm^2^ for the remaining 48 hours of culture in a 37°C incubator. For either the therapeutic (**Figure 5** and **6**) or prophylactic (**Figure S5D**) models, C57BL/6J mice were anesthetized using isoflurane and shaved on the right, rear flank on the days indicated in the figures. Each mouse received 5x10^4^ TC-1 cells in 100 µL of PBS injected with a 25G needle subcutaneously in the flank. Mice were subsequently monitored twice weekly for tumor volume and body weight. Prior to therapeutic treatment, all mice were randomized to ensure uniform tumor size distribution across groups and remove bias coming from varying tumor sizes. PBS or mouse AAC-E7 were injected RO at the indicated dose using the schedules shown in **Figures 5**, **S5** and **6**. For combination therapy studies, mice received an intraperitoneal injection of 5 mg/kg Cisplatin and 1x10^9^ AAC-E7 at the times indicated in **Figure 6A**. For re-challenge studies (**Figure S6A, B**), mice which had received the early Cisplatin treatment (day 7 and 9) were re-implanted with TC-1 tumors on day 73 and monitored as stated above until day 103. New, age matched C57BL/6J mice were also implanted on day 73 as a control and injected once with PBS. No additional treatment was administered. End-point criteria included total tumor volumes equal to or greater than 1,500 mm^3^ or a Body Condition Score of 2 or less. Tumor volumes were measured using calipers (Mitutoyo, Aurora, IL, USA) and calculated using the formula Volume = ½ (Length × Width^2^) where width is the smaller of the two measurements. In all tumor growth kinetics curves, except for spider plots, tumor volume values are plotted until the treatment group reached median survival. Each value is plotted as mean +/- SEM (standard error of means).

### Analysis of tumor infiltrating lymphocytes or splenocytes from TC-1 study

Tumors were dissected (23 days post implantation) from the right, rear flank and weighed. Tumors were minced using scissors into 2-4 mm pieces, then dissociated using a kit and gentleMACS Dissociator (Miltenyi Biotech) and incubated at 37°C for 45 minutes with continuous rotation. Single cell suspensions were also prepared from spleens and processed for flow cytometric analysis using BD Bioscience FACS lysis solution, Cytofix/Cytoperm kit, and eBioscience Foxp3/Transcription Factor Staining Buffer Set (Thermo Fisher Scientific). For *in vitro* restimulation, anti-CD28 (8 µg/mL, eBioscience) and E7_49-57_ (4 µg/mL, AnaSpec) was added for one hour and for an additional four hours in the presence of GolgiStop/GolgiPlug (BD Bioscience). Following restimulation, cells were stained for flow cytometry analysis. The panels are described in Supplementary Tables: ICS panel in **Table S13**, tetramer staining in **Table S14**, and Treg panel in **Table S15**.

### Statistical analysis

Statistical analyses were performed using GraphPad Prism 8.4 (San Diego, CA, USA). Data are plotted and stated in the text as mean ± standard deviation. Figure captions note whether an unpaired t test or one-way analysis of variance (ANOVA) was used. Evaluation of survival data in therapeutic tumor studies was performed using the Mantel-Cox test.

## Data availability statement

The original contributions presented in the study are included in the article/**Supplementary Material**. Further inquiries can be directed to the corresponding authors.

## Ethics statement

The animal studies were carried out according to protocols established by the American Association for Laboratory Animal Science and approved by the Institutional Animal Care and Use of Laboratory Animal Committee (IACUC) at SQZ Biotechnologies.

## Author contributions

Performed research: KB, CS, AR, LM, DS, ZT, APS, SK, AT, JM, AP, CP, DY. Designed research: KB, CS, AR, DS, ZT, APS, JM, ST, SL, KJS, HB, DY. Analyzed data: KB, CKS, AR, LM, DS, ZT, AS, SK, AT, DY. Supervision & Methodology: KB, AR, SL, KS, HB, DY. Contributed to the original concept, optimizations and/or chip design (experiments not necessarily included in the manuscript): KB, CS, AR, BW, RY, MD, LC, OAC, MM, DTB, JBG, KJS, AS, SL, HB, DY. Visualization: KB, CS, LM, SK. Wrote the manuscript: KB, CS, SL. Reviewed and edited the manuscript: KB, CS, SL, AS, HB. All authors contributed to the article and approved the submitted version.

## Acknowledgments

We would like to acknowledge and recognize the excellent animal care and assistance with the *in vivo* experiments by the department of laboratory animal research at SQZ Biotechnologies, Stephanie Westcott and Samantha-Jo Dilkes. We would like to thank Emrah Ilker Ozay for his help with the *in vivo* therapeutic combination tumor study, Aaron Handler for his contributions to the formulation of delivery material work, Ryan Stagg and Meg Yung for reviewing the manuscript and Brittany Stokes for her help with graphics.

## Conflict of interest

All listed authors are current or former SQZ Biotechnologies Company employees.

## Publisher’s note

All claims expressed in this article are solely those of the authors and do not necessarily represent those of their affiliated organizations, or those of the publisher, the editors and the reviewers. Any product that may be evaluated in this article, or claim that may be made by its manufacturer, is not guaranteed or endorsed by the publisher.

## Glossary

**Table:**

AAC	activating antigen carriers human RBCs squeeze processed with antigen and poly I:C adjuvant
AAC-E7	mouse RBCs squeeze processed with E7 HPV SLP antigen and poly I:C adjuvant
AACHPV	human RBCs squeeze processed on the research scale with E6 and E7 HPV SLP and poly I:C adjuvant
AAC-Ova	mouse RBCs squeeze processed with ovalbumin antigen and poly I:C adjuvant
AC	antigen carrier human RBCs squeeze processed with antigen (no adjuvant)
AC-E7	mouse RBCs squeeze processed with E7 HPV SLP antigen (no adjuvant)
AC-Ova	mouse RBCs squeeze processed with ovalbumin antigen (no adjuvant)
AF	Alexa Fluor
APC	antigen presenting cell
AT	adoptive transfer
AU	arbitrary units
CD	cluster of differentiation
cDC1	classical type 1 dendritic cell
CFSE	carboxyfluorescein succinimidyl ester
C-media	human RBCs squeeze processed with media (in the absence of antigen or adjuvant)
CMV	cytomegalovirus
C-poly I:C	human RBCs squeeze processed with poly I:C (no antigens)
CTLA-4	cytotoxic T-lymphocyte-associated protein 4
DC	dendritic cell
EC	empty carrier
FAM	5-carboxy-fluorescein
FOV	field of view
FSC	forward scatter
gMFI	geometric mean fluorescence intensity
HPV	human papilloma virus 16
ICS	intracellular cytokine staining
IFN	interferon
IL	interleukin
MoDC	monocyte derived dendritic cell
MOA	mechanism of action
NOAEL	no-observed-adverse-effect level
Ova	ovalbumin
PBS	phosphate-buffered saline
PBMC	Peripheral blood mononuclear cells
	
PD-1	programmed cell death protein 1
poly I:C	polyinosinic-polycytidylic
PS	phosphatidylserine
PSI	pound per square inch
RES	reticuloendothelial system
RBC	red blood cell
ROI	region of interest
RO	retro-orbital
RPM	red pulp macrophages
SLP	synthetic long peptide
SQZ-AAC-HPV	human RBCs squeeze processed on the clinical scale with E6 and E7 HPV SLP and poly I:C adjuvan
SSC	side scatter
TGF	transforming growth factor
TLR	toll-like receptor
