# Supplementary material for: Engineered red blood cells (activating antigen carriers) drive potent T cell responses and tumor regression in mice
Source: Front Immunol. 2022 Oct 3;13:1015585. doi: 10.3389/fimmu.2022.1015585 (PMC9573954; doi:10.3389/fimmu.2022.1015585)
Supplement: Supplementary file 1 [file DataSheet_1.docx]

Supplementary Material

# Supplementary Figures

## Supplementary Figure 1


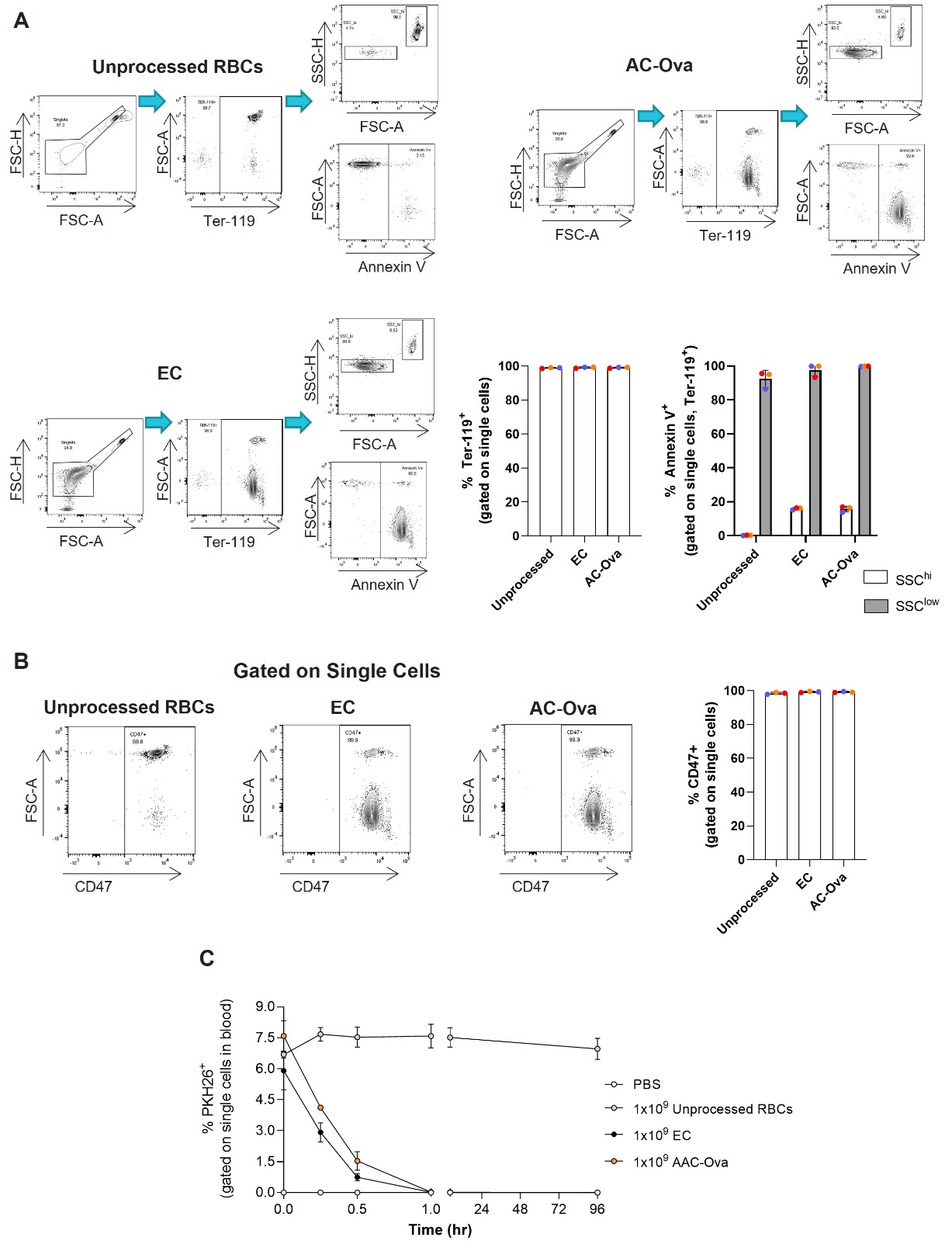


**Figure S1.** **Mouse carrier cell profiles and clearance. (A)** Representative gating strategies of unprocessed RBCs, media squeezed empty carriers (EC) and ovalbumin squeezed carriers (AC-Ova). Percent Ter-119^+^, Annexin V^+^ **(A)** and CD47^+^ **(B)** for each condition. **(C)** Clearance kinetics for unprocessed RBCs, EC and AAC-Ova (antigen and adjuvant). The clearance kinetics of the PBS treated group, the unprocessed RBC treated group, and the EC treated group are the same as shown in Fig. 1F. n = 2 independent studies.

## Supplementary Figure 2


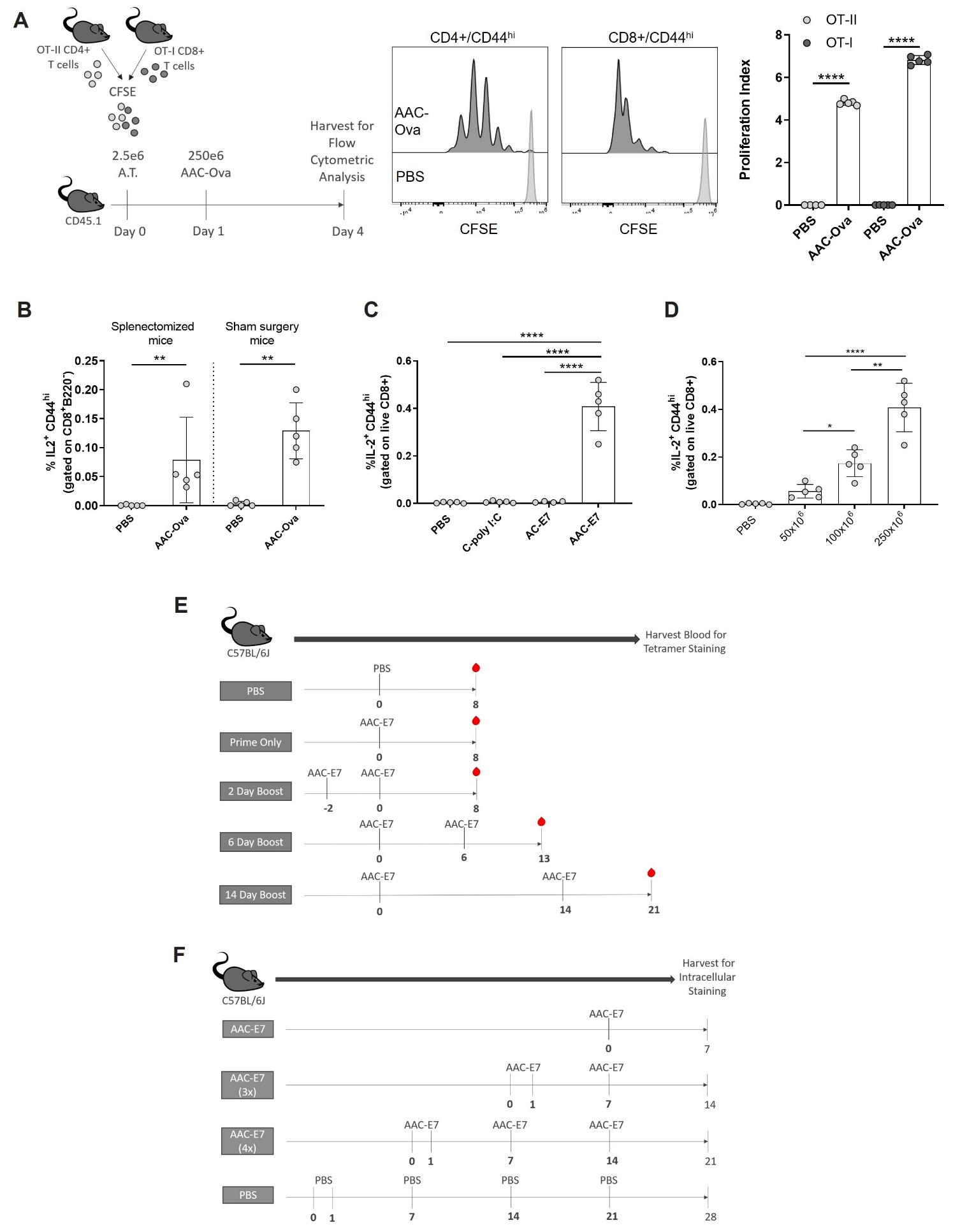


**Figure S2. CD4^+^/CD8^+^ T cell activation in endogenous AAC model. (A)** Scheme illustration of OT-I/OT-II adoptive transfer (AT) and AAC-Ova immunization with representative proliferation data. **(B)** CD8^+^ T cell IL-2 responses in the blood following PBS or AAC-Ova administration (250x10^6^/mouse) in splenectomized or sham surgery mice. **(C)** IL-2^+^ CD8^+^ T cells in C57BL/6J mice administered with PBS, C-poly I:C (adjuvant only), AC-E7 (antigen only), or AAC-E7 (antigen and adjuvant), 250x10^6^ carriers/mouse. **(D)** IL-2 responses in C57BL/6J mice administered with PBS or the indicated single dose of AAC-E7. Scheme illustration of **(E)** Blood tetramer staining: AAC-E7 boost study timeline for **Figure 2E** or **(F)** Endogenous response: AAC-E7 dose immunization timeline for **Figure 2F**. Figures show one dot per mouse for all studies. Unpaired t-test for A. One-way ANOVA for B-D. * P < 0.05, ** P < 0.005, **** P < 0.0001.

## Supplementary Figure 3


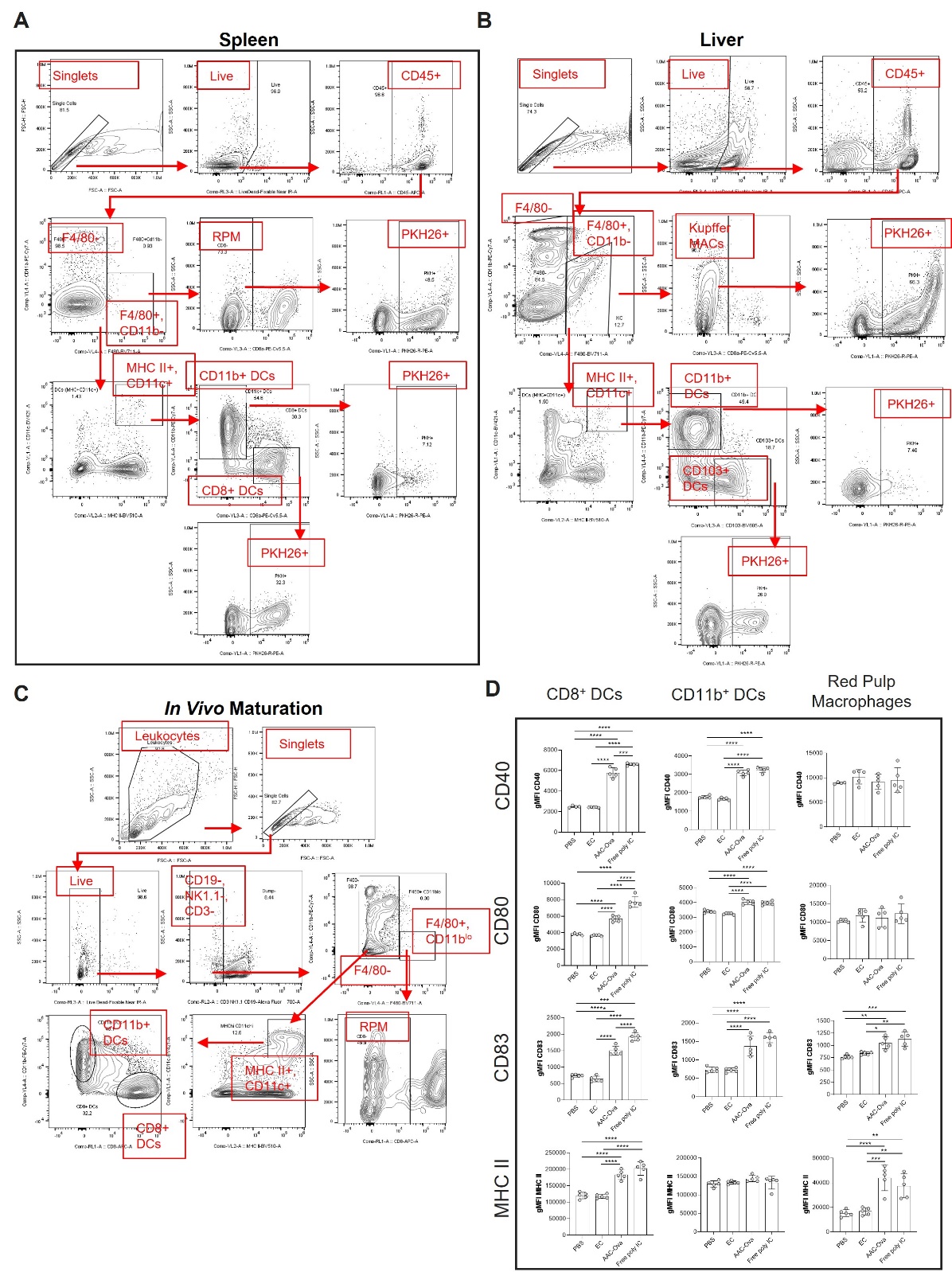


**Figure S3. Gating strategies for carrier cell uptake and APC maturation.** Representative gating strategies to demonstrate PKH26-labeled AAC-Ova uptake by APCs collected from recipient mouse **(A)** spleen or **(B)** liver. **(C)** Gating for APC maturation markers from recipient mouse spleens with **(D)** marker expression levels by gMFI. * P < 0.02, ** P < 0.05, *** P = 0.0001, **** P < 0.0001, one-way ANOVA.

## Supplementary Figure 4


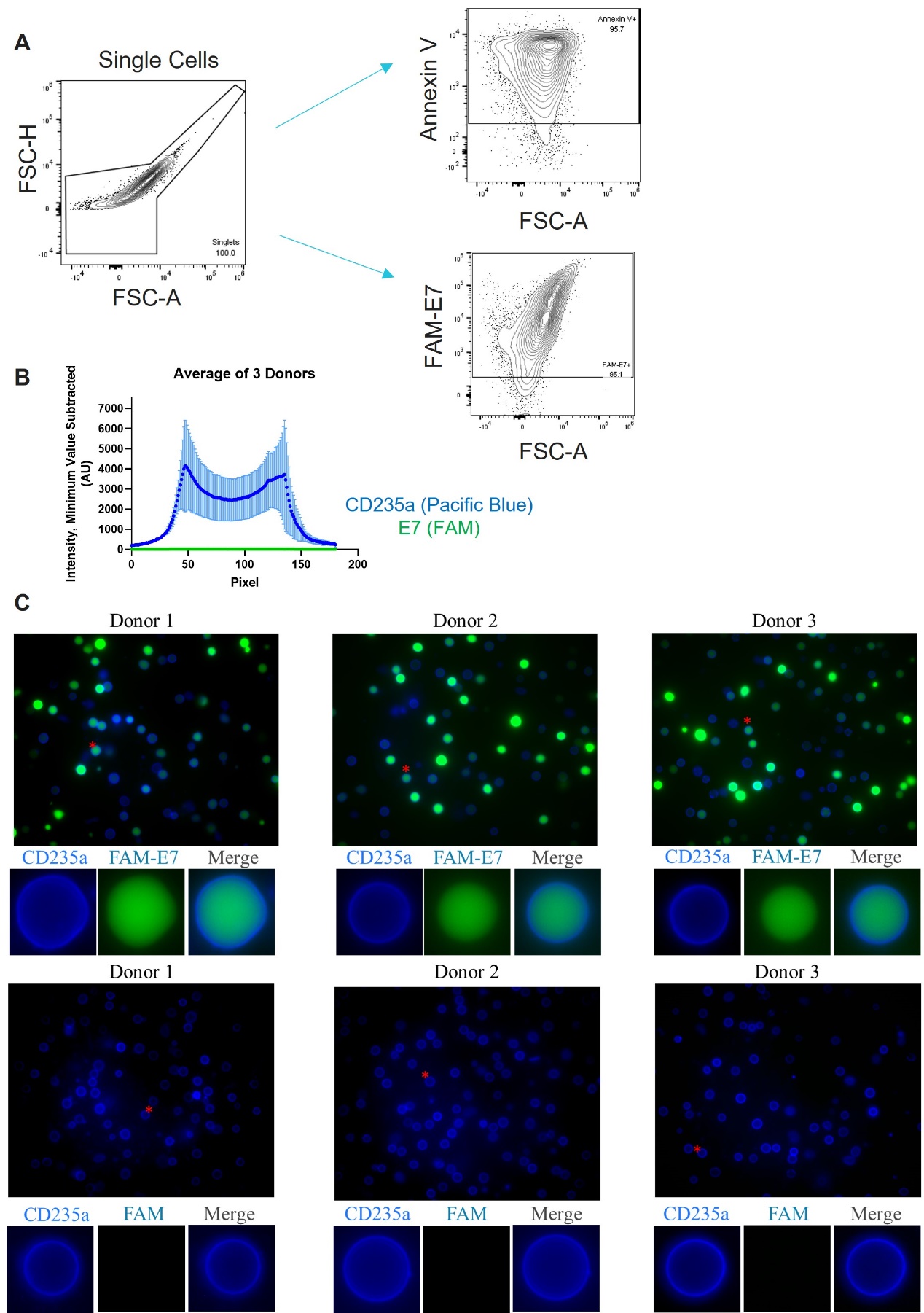


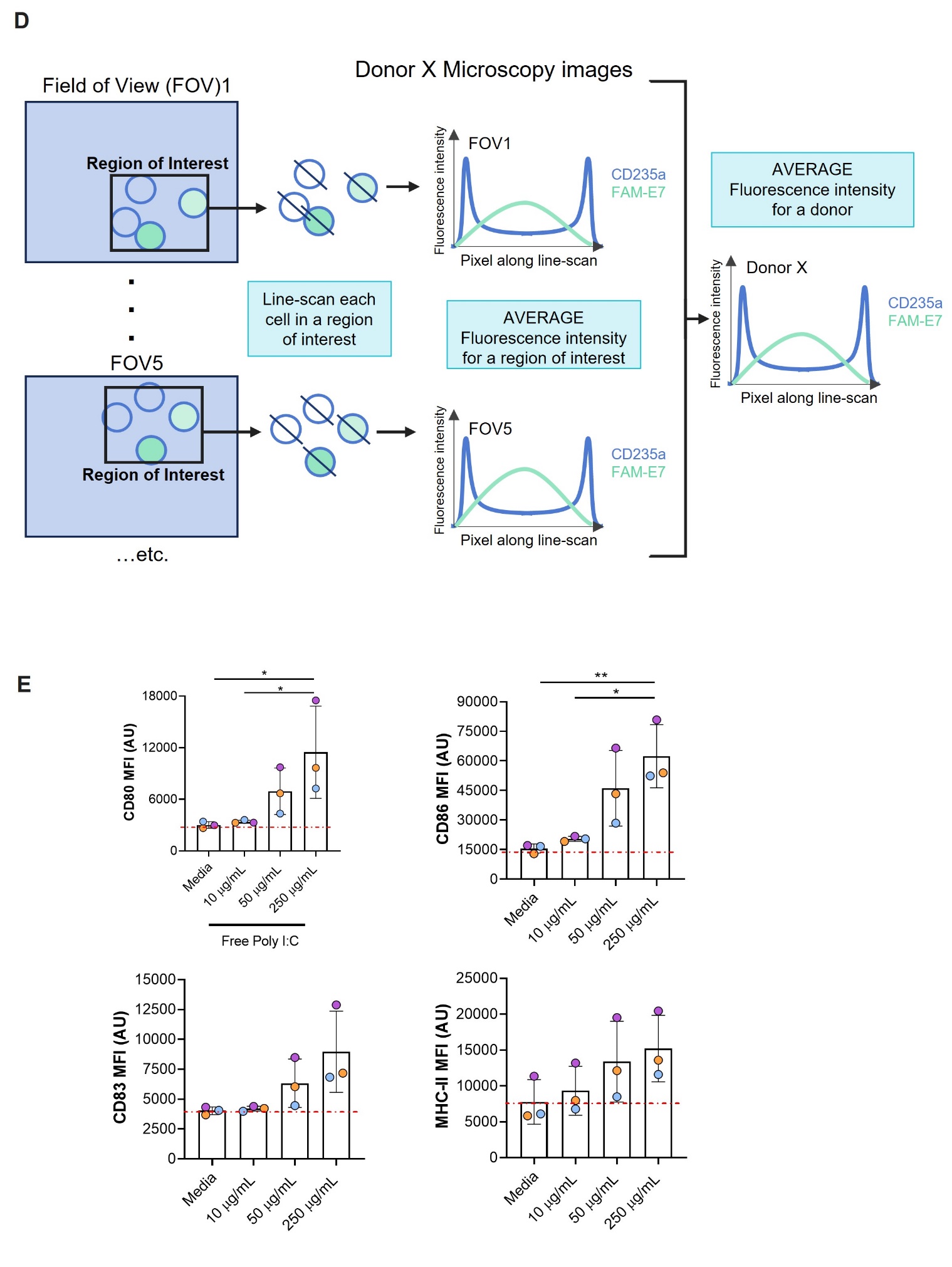

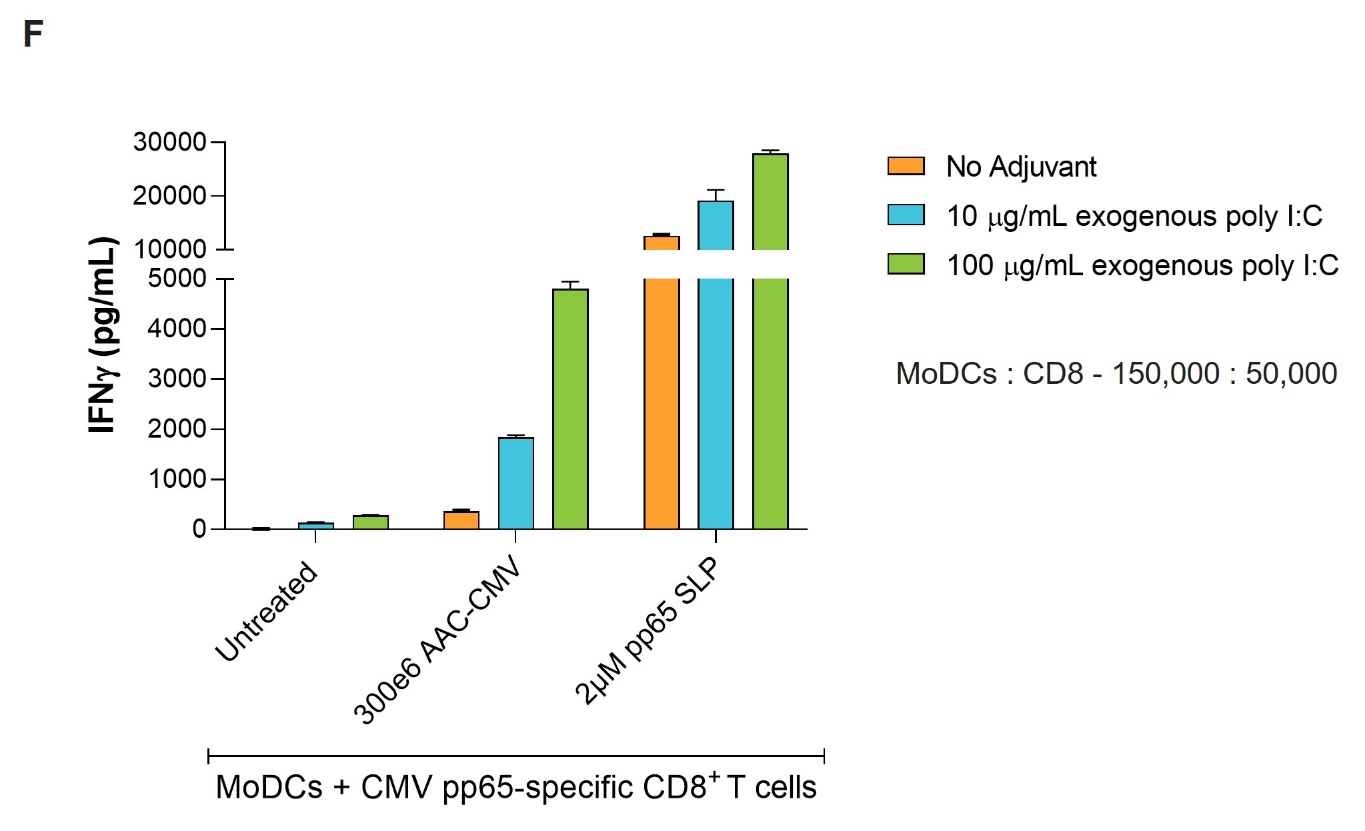


**Figure S4. Human carrier characterization and *in vitro* maturation of MoDCs.
(A)** Representative gating strategy showing human AACs squeeze processed with FAM-labeled E7 SLP, unlabeled E6, and poly I:C (1 mg/mL) and stained with Annexin V. Both the flow plots come from the single cell parent gate. **(B)** Graph showing the line-scans averaged across three different donors delivered with unlabeled E7, E6, and poly I:C and stained with CD235a. **(C)** Widefield representative fluorescent images for three different donors delivered with FAM-labeled E7 SLP (top) or unlabeled E7 SLP (bottom) in the presence of E6 SLP and poly I:C, stained for CD235a. Below each widefield image are examples of the merged channels for the AAC marked by a red asterisk. **(D)** Schematic depicting approach for image analysis to determine relative localization of FAM-E7 SLP and AAC membrane. **(E)** *In vitro* maturation and activation of MoDCs following 46 hour culture in the presence of 10-250 µg/mL of exogenous poly I:C. **(F)** Antigen-specific IFNγ responses following overnight cultures of human RBCs squeeze processed on the research scale with CMV pp65 SLP with HLA-A*02^+^ MoDCs and CMV pp65_495-503_-specific CD8^+^ T cells in the presence of exogenously added poly I:C. Supernatants were analyzed for IFNγ release by ELISA * P < 0.05, ** P < 0.01, one-way ANOVA.

## Supplementary Figure 5


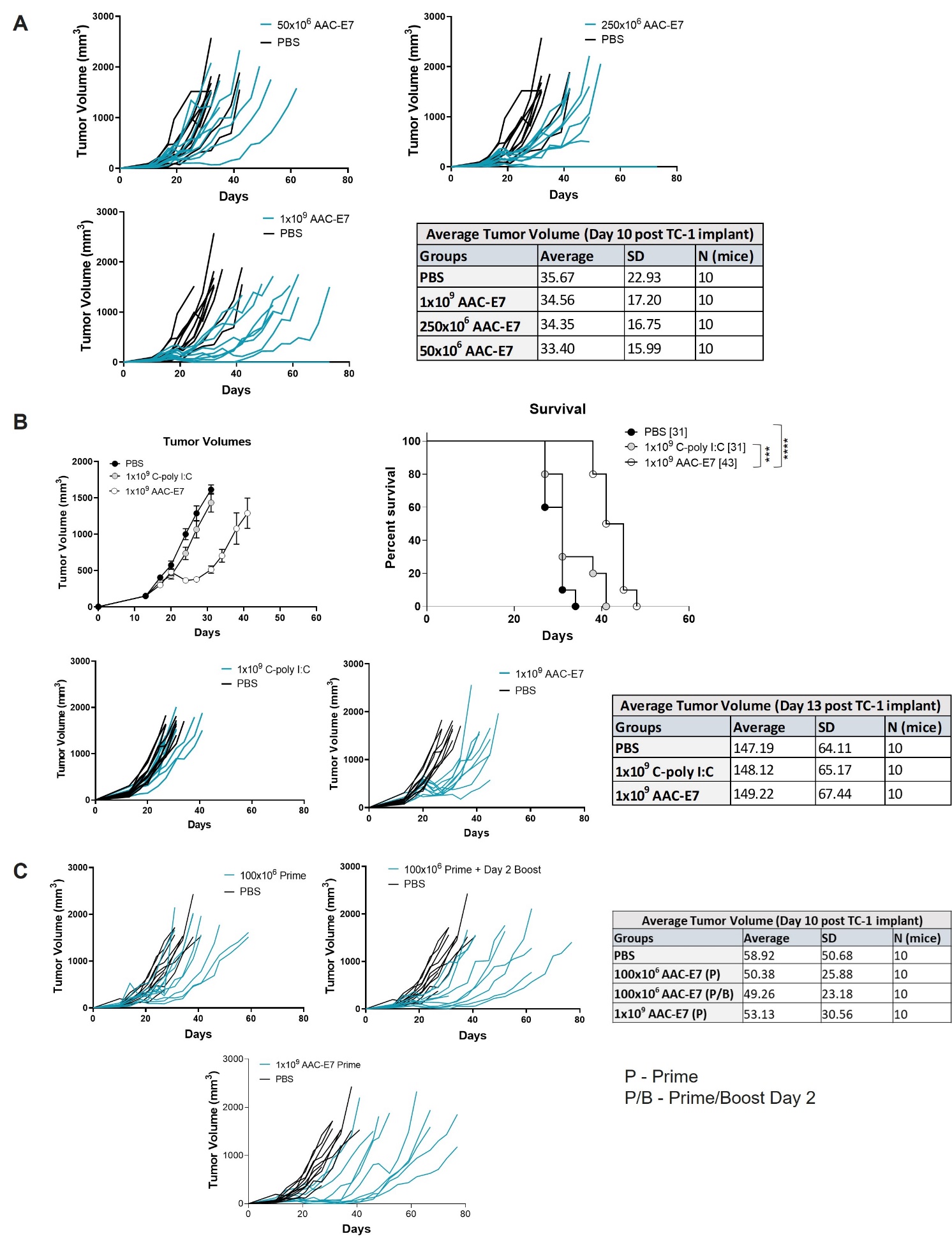


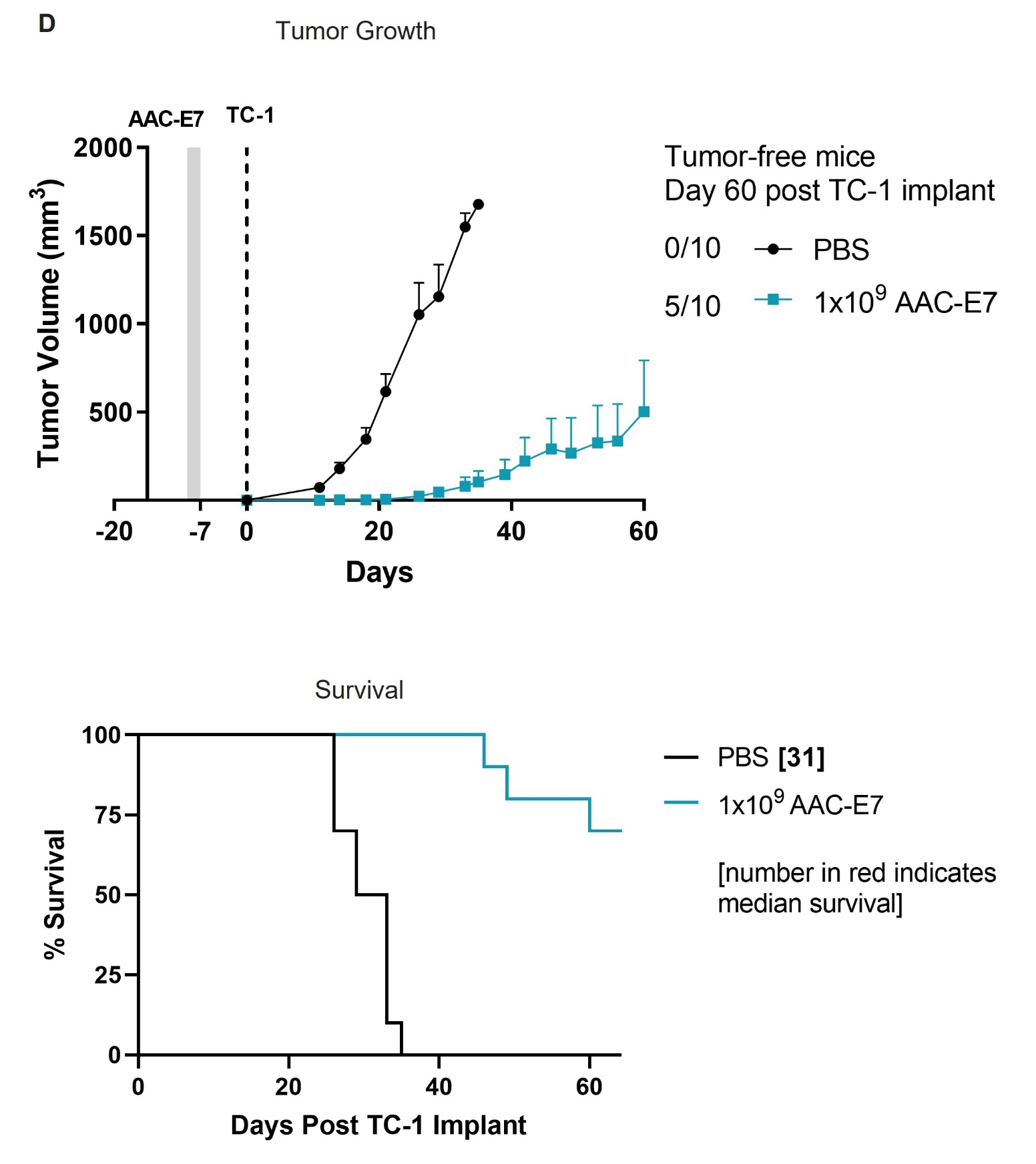

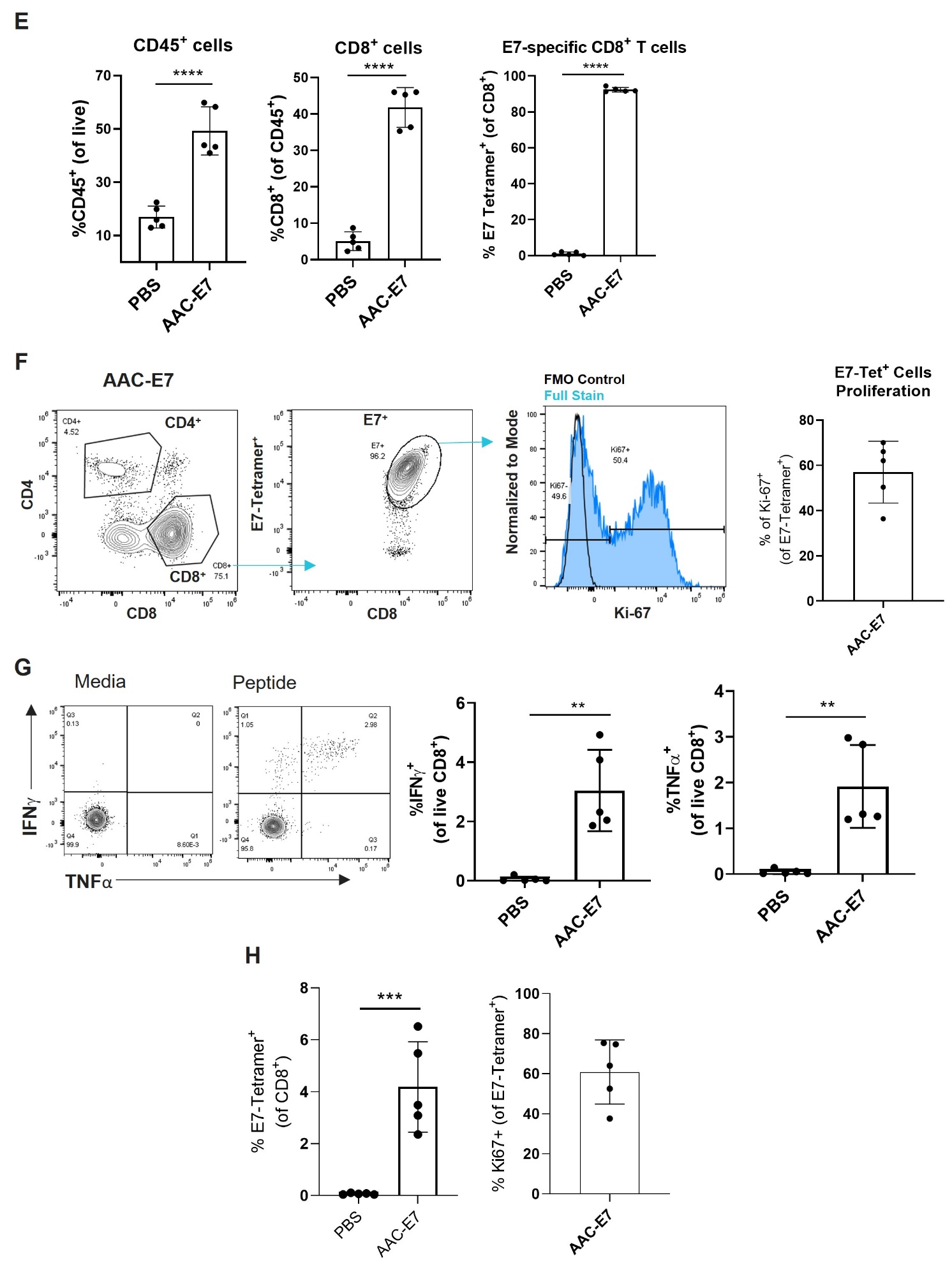


**Figure S5. Anti-tumor response and TIL analysis in tumor and periphery (A)** AAC-E7 dose response: spider plots and table of tumor volumes of TC-1 bearing mice before immunization. **(B)** Necessity of antigen in TC-1 model: (left) tumor growth curves and (right) median survival graph. Spider plots and table with tumor volumes of TC-1 bearing mice before immunization. **(C)** Effect of boost in TC-1 model: spider plots and table of tumor volumes of TC-1 bearing mice before immunization. **(D)** Tumor growth and survival in a prophylactic TC-1 model. **(E)** Percent tumor infiltrating CD45^+^, CD8^+^, or E7-specific cells. **(F)** Ki-67 staining of E7-specific tumor infiltrating CD8^+^ T cells from AAC-E7 immunized mouse. **(G) (Left)** Representative flow plot of AAC-E7 group: Percent of IFNγ^+^ and TNFα^+^ cells of live CD8^+^ T cells in spleens of TC-1 bearing mice following media or peptide restimulation. **(Right)** Percent of IFNγ^+^ and TNFα^+^ cells of CD8^+^ T cells following peptide restimulation. **(H)** Splenic E7-specific CD8^+^ T cells and Ki-67^+^ E7-tetramer^+^ CD8^+^ T cells. ** P < 0.01, *** P < 0.001, **** P < 0.0001, Mantel-Cox test for median survival and unpaired t-test for other figures. Poly I:C was squeezed at 1 mg/mL for all studies.

## Supplementary Figure 6


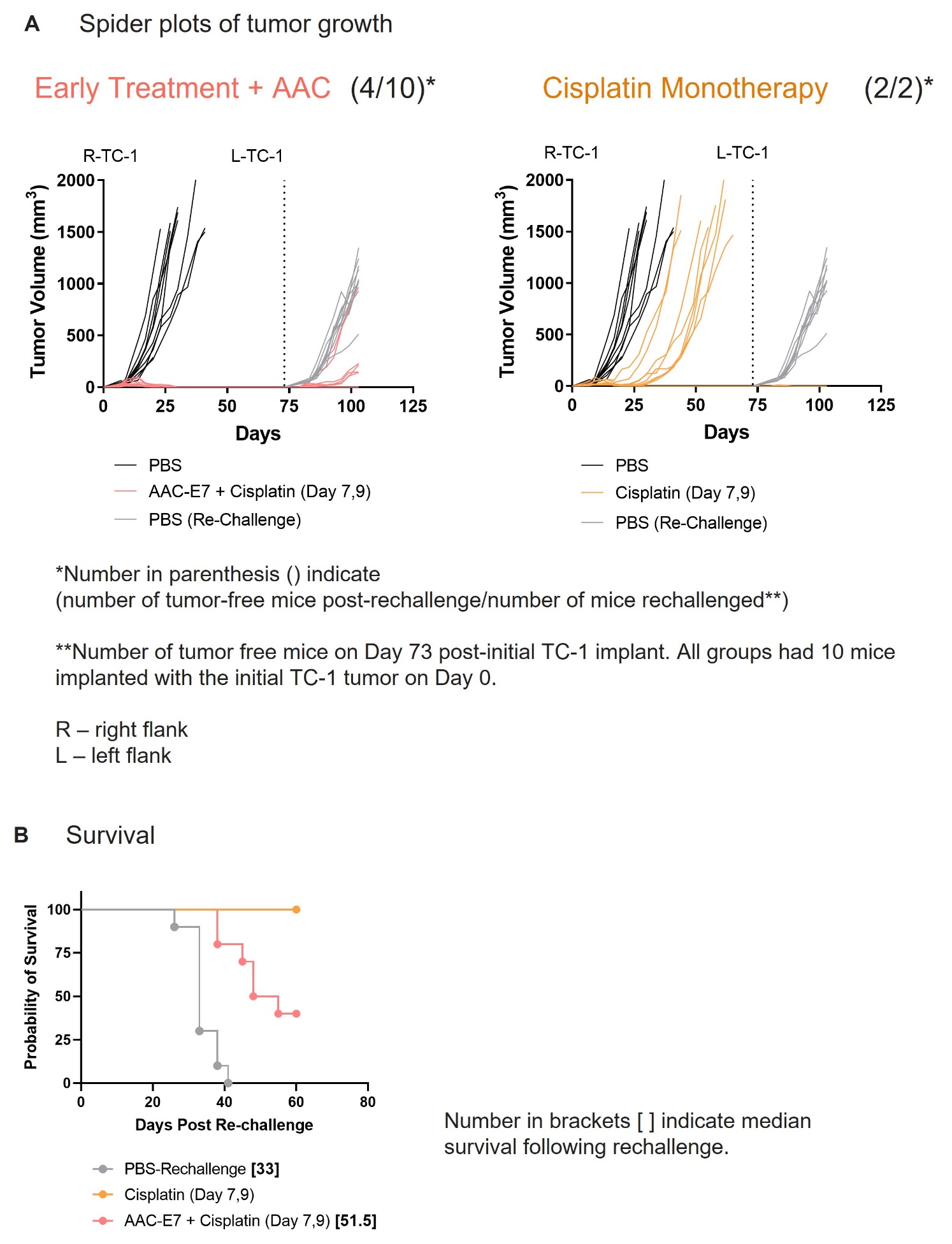


**Figure S6. Rechallenge of tumor-free mice in AAC-E7 and Cisplatin therapeutic combination.** On day 73 post-initial TC-1 implant, remaining tumor-free mice from the Cisplatin monotherapy and combination with AAC-E7 (from the study in Fig. 6B) were rechallenged with TC-1 cells on the opposite flank. All the animals from the PBS treated group from the initial TC-1 implant on the right flank (black line) reached endpoint by day 73. As a control, 10 age-matched mice were implanted with the TC-1 cells on the left-flank (gray line). **(A)** Spider plots for AAC-E7 and Cisplatin combination (left) and Cisplatin monotherapy (right). **(B)** Survival curve for rechallenged animals.

**AAC-E7 and Cisplatin “early” combination:**
Prior to rechallenge: 10 tumor-free animals
Post rechallenge: 4 tumor-free animals, 4 animals with slowed tumor growth, 2 animals similar to control animals

**Cisplatin monotherapy:**Prior to rechallenge: 2 tumor-free animals
Post rechallenge: 2 tumor-free animals
