# Supplementary material for: Engineered red blood cells (activating antigen carriers) drive potent T cell responses and tumor regression in mice
Source: Front Immunol. 2022 Oct 3;13:1015585. doi: 10.3389/fimmu.2022.1015585 (PMC9573954; doi:10.3389/fimmu.2022.1015585)
Supplement: Supplementary file 2 [file DataSheet_2.docx]

Supplementary Material

# Supplementary Table

## Supplementary Table 1

| **Name** | **Sequence** |
| --- | --- |
| mouse-E7 SLP | GQAEPDRAHYNIVTFSSKSDSTLRLSVQSTHVDIR |
| human-E7 SLP | QLCTELQTYMLDLQPETTYCKQQLL |
| E6 SLP | QLCTELQTTIHDIILECVYCKQQLL |
| FAM human-E7 SLP | QLCTELQTYMLDLQPETTYCKQQLL |
| CMV pp65 SLP | PPWQAGILARNLVPMVATVQGQNLKYQEFFWDAND |
| E7_49 –57_ | RAHYNIVTF |
| E7_11-20_ | YMLDLQPETT |
| Ova_257-264_ | SIINFEKL |

**Table S1. Peptide sequences**

## Supplementary Table 2

| Type of Study  (GLP status) | Species/Strain | Method of Administration | Dose^a^:  AAC-E7 (#/kg/dose) /  Poly I:C (μg/kg/dose) | Gender and n per Group |
| --- | --- | --- | --- | --- |
| 22-Day Repeated Dose Toxicity Study of AAC-E7 with 30-Day Recovery (GLP) | C57Bl/6J mice | Intravenous; Day 1, 2, 8, 15, 22 | Control: 0/0  High^b,c^: 14 x10^9^ / 330 | Main Study:  20/sex/group  Recovery:  8/sex/group |

**Table S2. Nonclinical Toxicity Studies of AAC-E7**

^a^Poly I:C is dosed as part of the dose of AAC-E7.

^b^The AAC-E7 dose shown is based on a calculation assuming a 23 g mouse body weight and the average dose across the 5 days of dosing.

^c^The poly I:C dose shown is based on a calculation assuming a 23 g mouse body weight, the average concentration of the poly I:C (μg/mL) across the 5 days of dosing, and the volumes dosed (50 μL/mouse and 170 μL/mouse for the low and high dose group, respectively).

A GLP safety study of AAC-E7 was conducted in mice. The study was performed in accordance with the U.S. Department of Health and Human Services, Food and Drug Administration, United States Code of Federal Regulations, Title 21, Part 58: Good Laboratory Practice for Nonclinical Laboratory Studies and as accepted by Regulatory Authorities throughout the European Union (OECD Principles of Good Laboratory Practice), Japan (MHLW), and other countries that are signatories to the OECD Mutual Acceptance of Data Agreement.

The study design is shown in above Supplementary Table 2. C57Bl/6J mice received intravenous tail vein injections of AAC-E7 on Day 1, Day 2, Day 8, Day 15, and Day 22, followed by a 30-day recovery period in some animals at two different dose levels. PBS was used as the control.

The study was conducted at a third-party FDA registered contract research organization. The following parameters and end points were evaluated in this study: clinical signs, body weights, body weight gains, food consumption, clinical pathology parameters (hematology and clinical chemistry), gross necropsy findings, organ weights, and histopathologic examinations. All evaluations including histopathological, clinical chemistry and hematology were independently conducted by toxicologists or board-certified pathologists at the CRO. The findings are summarized below:

| **Parameter** | **Finding** |
| --- | --- |
| Body weight changes | None |
| Food consumption changes | None |
| Clinical signs | None |
| Hematology | No significant changes |
| Clinical chemistry | No significant changes |
| Gross necropsy findings | None |
| Organ weight changes | Spleen 20% increase low dose and 24% high dose on day 23 and normal after 30 days of recovery |
| Histopathology | No significant findings |

The no-observed-adverse-effect level (NOAEL) of mouse AAC-E7 intravenously administered to C57BL/6J mice was considered to be ~14 E9 AACs/kg/dose (High dose group; average poly I:C ~330 μg/kg/dose). The full safety study report has been submitted and reviewed by the regulatory authorities in the US, Europe and Asia.

Detailed summaries of hematology and clinical chemistry findings for male and female animals treated with the high AAC dose or the control group (no treatment) are provided in the tables below for the last day of day of the study (day 52).

**Summary** **of** **Hematology** **Values**

| **Sex: Male** | | **WBC**  (10^3/uL) | **NEUT**  (10^3/uL) | **LYMPH**  (10^3/uL) | **MONO**  (10^3/uL) |
| --- | --- | --- | --- | --- | --- |
| **0** | Mean | 6.110 | 0.230 | 5.580 | 0.160 |
| cell no./  kg/dose | SD | - | - | - | - |
|  | Number of Animals | 1 | 1 | 1 | 1 |
| **Group 1 (No treatment)** |  | - | - | - | - |
| **13.2e9** | Mean | 5.797 | 0.267 | 5.230 | 0.167 |
| cell no./  kg/dose | SD | 0.856 | 0.040 | 0.799 | 0.032 |
|  | Number of Animals | 3 | 3 | 3 | 3 |
| **Group 3 (High AAC dose)** | tCtrl | 0.95 | 1.16 | 0.94 | 1.04 |

| **Sex: Female** | | **WBC**  (10^3/uL) | **NEUT**  (10^3/uL) | **LYMPH**  (10^3/uL) | **MONO**  (10^3/uL) |
| --- | --- | --- | --- | --- | --- |
| **0**  cell no./  kg/dose | Mean | 2.243 | 0.123 | 1.980 | 0.060 |
|  | SD | 0.486 | 0.022 | 0.446 | 0.026 |
|  | Number of Animals | 4 | 4 | 4 | 4 |
| **Group (No treatment)** |  | - | - | - | - |
| **13.2e9** | Mean | 1.773 | 0.105 | 1.578 | 0.043 |
| cell no./ | SD | 0.488 | 0.053 | 0.409 | 0.024 |
| kg/dose | Number of Animals | 4 | 4 | 4 | 4 |
| **Group 3 (High AAC dose)** | tCtrl | 0.79 | 0.86 | 0.80 | 0.71 |

| **Sex: Male** | | **RBC**  (10^6/uL) | **HGB**  (g/dL) | **HCT**  (%) | **RDW**  (%) |
| --- | --- | --- | --- | --- | --- |
| **0** | Mean | 10.000 | 14.20 | 44.40 | 14.80 |
| cell no./ | SD | - | - | - | - |
| kg/dose | Number of Animals | 1 | 1 | 1 | 1 |
| **Group 1** **(No treatment)** |  | - | - | - | - |
| **13.2e9** | Mean | 9.550 | 13.83 | 43.07 | 15.07 |
| cell no./ | SD | 0.248 | 0.32 | 0.67 | 0.21 |
| kg/dose | Number of Animals | 3 | 3 | 3 | 3 |
| **Group 3** **(High AAC dose)** | tCtrl | 0.96 | 0.97 | 0.97 | 1.02 |

| **Sex: Female** | | **RBC**  (10^6/uL) [G] | **HGB**  (g/dL) [G] | **HCT**  (%) [G] | **RDW**  (%) [G] |
| --- | --- | --- | --- | --- | --- |
| **0**  cell no./  kg/dose | Mean | 9.773 | 14.00 | 43.58 | 15.53 |
|  | SD | 0.393 | 0.48 | 1.04 | 0.26 |
|  | Number of Animals | 4 | 4 | 4 | 4 |
| **Group 1 (No treatment)** |  | - | - | - | - |
| **13.2e9** | Mean | 9.310 | 13.55 | 41.48 | 15.68 |
| cell no./ | SD | 0.278 | 0.37 | 1.48 | 0.29 |
| kg/dose | Number of Animals | 4 | 4 | 4 | 4 |
| **Group 3 (High AAC dose)** | tCtrl | 0.95 | 0.97 | 0.95 | 1.01 |

| **Sex: Male** | | **Clinical Chemistry** | | | | | | |
| --- | --- | --- | --- | --- | --- | --- | --- | --- |
|  |  | UREAN  (mg/dL) | CREAT  (mg/dL) | GLUC  (mg/dL) | CHOL  (mg/dL) | TRIG  (mg/dL) | TPROT  (g/dL) | ALB  (g/dL) |
| **0** | Mean | 0.23 | 0.20 | 350.3 | 121.0 | 90.0 | 4.67 | 3.50 |
| cell no./ | SD | 1.00 | 0.00 | 13.3 | 9.6 | 29.7 | 0.15 | 0.10 |
| kg/dose | Number of Animals | 3 | 3 | 3 | 3 | 3 | 3 | 3 |
| **Group 1 (No treatment)** |  | - | - | - | - | - | - | - |
| **13.2e9** | Mean | 22.0 | 0.20 | 334.8 | 123.0 | 98.5 | 4.75 | 3.55 |
| cell no./ | SD | 2.7 | 0.08 | 25.1 | 13.5 | 18.8 | 0.13 | 0.13 |
| kg/dose | Number of Animals | 4 | 4 | 4 | 4 | 4 | 4 | 4 |
| **Group 3 (High AAC dose)** | tCtrl | 0.96 | 1.00 | 0.96 | 1.02 | 1.09 | 1.02 | 1.01 |

| **Sex: Male** | | **Clinical Chemistry** | | | | | | |
| --- | --- | --- | --- | --- | --- | --- | --- | --- |
|  |  | GLOB  (g/dL) | A/G  (ratio) | CA  (mg/dL) | PHOS  (mg/dL) | NA  (mEq/L) | K  (mEq/L) | CL  (mEq/L) |
| **0**  cell no./  kg/dose  **Group 1 (No treatment)** | Mean | 1.17 | 3.03 | 10.00 | 7.97 | 144.7 | 5.35 | 106.9 |
|  | SD | 0.12 | 0.31 | 0.26 | 0.35 | 1.5 | 0.40 | 1.0 |
|  | Number of Animals | 3 | 3 | 3 | 3 | 3 | 3 | 3 |
|  |  | - | - | - | - | - | - | - |
| **13.2e9** | Mean | 1.20 | 2.98 | 9.80 | 6.80 | 144.5 | 5.22 | 107.9 |
| cell no./ | SD | 0.08 | 0.28 | 0.14 | 0.98 | 2.4 | 0.66 | 1.0 |
| kg/dose | N | 4 | 4 | 4 | 4 | 4 | 4 | 4 |
| **Group 3 (High AAC dose)** | tCtrl | 1.03 | 0.98 | 0.98 | 0.85 | 1.00 | 0.98 | 1.01 |

| **Sex: Female** | | **Clinical Chemistry** | | | | | | |
| --- | --- | --- | --- | --- | --- | --- | --- | --- |
|  |  | AST  (U/L) | ALT  (U/L) | ALP  (U/L) | GGT  (U/L) | LDH  (U/L) | CK  (U/L) | TBIL  (mg/dL) |
| **0** | Mean | 37.8 | 20.5 | 104.3 | 1.5 | 108.8 | 37.5 | 0.050 |
| cell no./ | SD | 3.8 | 1.3 | 10.7 | 0.0 | 8.3 | 3.3 | 0.037 |
| kg/dose | Number of Animals | 4 | 4 | 4 | 4 | 4 | 4 | 4 |
| **Group 1 (No treatment)** |  | - | - | - | - | - | - | - |
| **13.2e9** | Mean | 37.5 | 19.5 | 107.3 | 1.5 | 123.3 | 38.5 | 0.065 |
| cell no./ | SD | 2.1 | 2.4 | 10.6 | 0.0 | 30.6 | 4.5 | 0.006 |
| kg/dose | Number of Animals | 4 | 4 | 4 | 4 | 4 | 4 | 4 |
| **Group 3 (High AAC dose)** | tCtrl | 0.99 | 0.95 | 1.03 | 1.00 | 1.13 | 1.03 | 1.30 |

| **Sex: Female** | | **Clinical Chemistry** | | | | | | |
| --- | --- | --- | --- | --- | --- | --- | --- | --- |
|  |  | UREAN  (mg/dL) | CREAT  (mg/dL) | GLUC  (mg/dL) | CHOL  (mg/dL) | TRIG  (mg/dL) | TPROT  (g/dL) | ALB  (g/dL) |
| **0** | Mean | 19.0 | 0.18 | 298.0 | 88.8 | 114.8 | 4.70 | 3.60 |
| cell no./ | SD | 3.7 | 0.05 | 6.1 | 9.7 | 19.6 | 0.22 | 0.08 |
| kg/dose | Number of Animals | 4 | 4 | 4 | 4 | 4 | 4 | 4 |
| **Group 1 (No treatment)** |  | - | - | - | - | - | - | - |
| **13.2e9** | Mean | 19.8 | 0.15 | 269.8 | 72.5 | 89.8 | 4.63 | 3.70 |
| cell no./ | SD | 1.5 | 0.06 | 17.3 | 3.7 | 23.5 | 0.17 | 0.08 |
| kg/dose | Number of Animals | 4 | 4 | 4 | 4 | 4 | 4 | 4 |
| **Group 3 (High AAC dose)** | tCtrl | 1.04 | 0.86 | 0.91 | 0.82 | 0.78 | 0.98 | 1.03 |

A summary of the microscopic findings for the control group and the high dose group on the last day of the study are provided in the table below.

**Summary of Microscopic Pathology on last day of the study**

| **Microscopic Pathology** | | | | |
| --- | --- | --- | --- | --- |
| **Summary: Incidence**  **Number of Animals:** | **Male** | | **Female** | |
|  | **0**  cell no./ kg/dose  **Group 1 (No treatment)** | **13.2e9**  cell no./ kg/dose  **Group 3 (High AAC dose)** | **0**  cell no./ kg/dose  **Group 1 (No treatment)** | **13.2e9**  cell no./ kg/dose  **Group 3 (High AAC dose)** |
|  | **8** | **8** | **8** | **8** |
| **BONE MARROW, STERNUM** | | | | |
| Examined | 8 | 8 | 8 | 8 |
| No Visible Lesions | 8 | 8 | 8 | 8 |
| **BRAIN** | | | | |
| Examined | 8 | 8 | 8 | 8 |
| No Visible Lesions | 7 | 8 | 8 | 8 |
| Cyst; squamous | 1 | 0 | 0 | 0 |
| **HEART** | | | | |
| Examined | 8 | 8 | 8 | 8 |
| No Visible Lesions | 8 | 8 | 8 | 8 |
| **KIDNEY** | | | | |
| Examined | 8 | 8 | 8 | 8 |
| No Visible Lesions | 6 | 6 | 6 | 7 |
| Cast | 1 | 1 | 1 | 1 |
| .... minimal | 1 | 1 | 1 | 1 |
| Infiltration, mononuclear cell; interstitial | 0 | 0 | 0 | 1 |
| .... minimal | 0 | 0 | 0 | 1 |
| Cyst | 1 | 0 | 0 | 0 |
| Basophilia; tubular | 0 | 1 | 0 | 0 |
| .... minimal | 0 | 1 | 0 | 0 |
| Chronic progressive nephropathy | 0 | 0 | 1 | 0 |
| .... minimal | 0 | 0 | 1 | 0 |
| **LARGE INTESTINE, COLON** | | | | |
| Examined | 8 | 8 | 8 | 7 |
| No Visible Lesions | 8 | 8 | 8 | 7 |
| Not Examined: Not Present In Section. | . | . | 0 | 1 |
| **LIVER** | | | | |
| Examined | 8 | 8 | 8 | 8 |
| No Visible Lesions | 2 | 4 | 7 | 5 |
| Infiltration, mixed cell | 6 | 4 | 1 | 3 |
| .... minimal | 6 | 4 | 1 | 3 |
| **LUNG** | | | | |
| Examined | 8 | 8 | 8 | 8 |
| No Visible Lesions | 8 | 7 | 7 | 7 |
| Bronchioloalveolar adenoma | 0 | 0 | 0 | 1 |
| Hemorrhage | 0 | 1 | 0 | 0 |
| .... minimal | 0 | 1 | 0 | 0 |
| Infiltration, mixed cell | 0 | 0 | 1 | 0 |
| .... minimal | 0 | 0 | 1 | 0 |
| **LYMPH NODE, MANDIBULAR** | | | | |
| Examined | 8 | 8 | 8 | 8 |
| No Visible Lesions | 8 | 8 | 8 | 8 |
| **LYMPH NODE, MESENTERIC** | | | | |
| Examined | 8 | 8 | 8 | 6 |
| No Visible Lesions | 8 | 8 | 7 | 6 |
| Not Examined: Insufficient Tissue Available For | . | . | 0 | 1 |
| Evaluation. |  |  |  |  |
| Not Examined: Not Present In Section. | . | . | 0 | 1 |
| Aggregate; increased, macrophage | 0 | 0 | 1 | 0 |
| .... mild | 0 | 0 | 1 | 0 |
| **OVARY** | | | | |
| Examined | . | . | 8 | 8 |
| No Visible Lesions | . | . | 8 | 8 |
| **SMALL INTESTINE, DUODENUM** | | | | |
| Examined | 8 | 8 | 8 | 8 |
| No Visible Lesions | 8 | 8 | 8 | 8 |
| **SMALL INTESTINE, ILEUM** | | | | |
| Examined | 8 | 8 | 8 | 7 |
| No Visible Lesions | 8 | 8 | 8 | 7 |
| Not Examined: Not Present In Section. | . | . | 0 | 1 |
| **SMALL INTESTINE, JEJUNUM** | | | | |
| Examined | 8 | 8 | 8 | 8 |
| No Visible Lesions | 8 | 8 | 8 | 8 |
| **SPLEEN** | | | | |
| Examined | 8 | 8 | 8 | 8 |
| No Visible Lesions | 8 | 8 | 4 | 6 |
| Pigmented macrophage; red pulp | 0 | 0 | 4 | 2 |
| .... minimal | 0 | 0 | 4 | 2 |
| **STOMACH** | | | | |
| Examined | 8 | 8 | 8 | 8 |
| No Visible Lesions | 8 | 8 | 8 | 8 |
| **TESTIS** | | | | |
| Examined | 8 | 8 | . | . |
| No Visible Lesions | 8 | 7 | . | . |
| Degeneration/atrophy; tubular | 0 | 1 | . | . |
| .... minimal | 0 | 1 | . | . |

## Supplementary Table 3

| **Mouse Carrier Characterization:** | | | | | | |
| --- | --- | --- | --- | --- | --- | --- |
| **Target** | | **Label** | | **Company** | | **Catalog Number** |
| CD47 | | Brilliant Violet 421 | | Biolegend | | 127527 |
| Isotype IgG2b (instead of CD47) | | Brilliant Violet 421 | | Biolegend | | 400640 |
| Annexin V | | FITC | | Biolegend | | 640945 |
| Ter-119 | | PE | | Biolegend | | 116208 |
| **Human Carrier Characterization:** | | | | | | |
| **Target** | **Label** | | **Company** | | **Catalog Number** | |
| Annexin V | Alexa Fluor 647 | | Biolegend | | 640943 | |

**Table S3. *In vitro* characterization of carriers**

## Supplementary Table 4

| **Target** | **Label** | **Company** | **Catalog Number** |
| --- | --- | --- | --- |
| CD11c | Brilliant Violet 421 | Biolegend | 117343 |
| I-A/I-E (MHC class II) | Brilliant Violet 510 | Biolegend | 107636 |
| CD103 | Brilliant Violet 605 | Biolegend | 121433 |
| F4/80 | Brilliant Violet 711 | Biolegend | 123147 |
| CD19 | Alexa Fluor 700 | Biolegend | 115528 |
| NK-1.1 | Alexa Fluor 700 | Biolegend | 108730 |
| LIVE/DEAD Fixable | Near-IR | ThermoFisher Scientific | L10119 |
| CD45 | APC | Biolegend | 103112 |
| CD8a | PE/Cy 5.5 | Biolegend | 100710 |
| CD11b | PE/Cy7 | BioLegend | 101216 |

**Table S4. Assessment of PKH26-labeled AAC-Ova uptake *in vivo***

## Supplementary Table 5

| **Target** | **Label** | **Company** | **Catalog Number** |
| --- | --- | --- | --- |
| **Panel 1** | | | |
| CD40 | PerCp Cy5.5 | Biolegend | 124624 |
| CD86 | PE | Biolegend | 105008 |
| **Panel 2** | | | |
| CD80 | PerCp Cy5.5 | Biolegend | 104722 |
| CD83 | PE | Biolegend | 121508 |
| **Common to both panels 1 and 2** | | | |
| F4/80 | Brilliant Violet 711 | Biolegend | 123147 |
| I-A/I-E (MHC class II) | Brilliant Violet 510 | Biolegend | 107636 |
| CD11c | Brilliant Violet 421 | Biolegend | 117343 |
| CD8a | APC | Biolegend | 100712 |
| LIVE/DEAD Fixable | Near-IR | ThermoFisher Scientific | L10119 |
| CD19 | Alexa Fluor 700 | Biolegend | 115528 |
| NK1.1 | Ax700 | Biolegend | 108730 |
| CD3 | Ax700 | Biolegend | 100216 |
| CD11b | PE/Cy7 | Biolegend | 101216 |

**Table S5. Endogenous APC maturation**

## Supplementary Table 6

| **Target** | **Label** | **Company** | **Catalog Number** |
| --- | --- | --- | --- |
| CD8a | Pacific Blue | BioLegend | 100725 |
| IFNγ | Brilliant Violet 605 | BioLegend | 505840 |
| CD44 | Alexa Fluor 700 | ThermoFisher Scientific | 56-0441-82 |
| LIVE/DEAD Fixable | Near-IR | ThermoFisher Scientific | L10119 |
| CD45R/B220 | FITC | BioLegend | 103206 |
| CD11b | FITC | BioLegend | 101206 |
| IL-2 | PE | BioLegend | 503808 |
| CD4 | PE/Cy7 | BioLegend | 100528 |

**Table S6. Endogenous responses- ICS**

## Supplementary Table 7

| **Target** | **Label** | **Company** | **Catalog Number** |
| --- | --- | --- | --- |
| CD4 | Brilliant Violet 510 | BioLegend | 100559 |
| B220 | Alexa Fluor700 | BioLegend | 103232 |
| LIVE/DEAD Fixable | Near-IR | Thermo Fisher Scientific | L10119 |
| E7 Tetramer (H-2Db HPV-16 E7, RAHYNIVTF) | APC | MBL International | TB-5008-2 |
| CD8 | FITC | BioLegend | 100706 |
| CD3e | PE | BioLegend | 100308 |
| CD44 | PE/Cy7 | BioLegend | 103030 |

**Table S7. Endogenous responses-tetramer staining**

## Supplementary Table 8

| **Target** | **Label** | **Company** | **Catalog Number** |
| --- | --- | --- | --- |
| CD45R/B220 | Brilliant Violet 421 | BioLegend | 103240 |
| CD11b | Brilliant Violet 421 | BioLegend | 101236 |
| CD45.1 | Brilliant Violet 605 | BioLegend | 110738 |
| LIVE/DEAD Fixable | Near-IR | ThermoFisher Scientific | L10119 |
| CD44 | APC | BioLegend | 103012 |
| CD4 | PerCP-Cy5.5 | BioLegend | 100434 |
| CD45.2 | PE | BioLegend | 109808 |
| CD8a | PE/Cy7 | BioLegend | 100722 |

**Table S8. Proliferation in OT-I/OT-II model**

## Supplementary Table 9

| **Target** | **Label** | **Company** | **Catalog Number** |
| --- | --- | --- | --- |
| CD3 | Pacific Blue | BioLegend | 344824 |
| LIVE/DEAD Fixable | Near-IR | ThermoFisher Scientific | L10119 |
| HLA-A2 | APC | BioLegend | 343308 |
| CD45 | APC/Cy7 | BioLegend | 304014 |
| CD14 | Alexa Fluor 488 | BioLegend | 301811 |
| CD19 | PE/Cy7 | BioLegend | 363012 |

**Table S9. Human monocyte purity**

## Supplementary Table 10

| **Target** | **Label** | **Company** | **Catalog Number** |
| --- | --- | --- | --- |
| CD80 | Brilliant Violet 421 | BioLegend | 305222 |
| HLA-DR | Brilliant Violet 605 | BioLegend | 307640 |
| LIVE/DEAD Fixable | Near-IR | ThermoFisher Scientific | L10119 |
| CD11c | APC | BioLegend | 337208 |
| CD14 | Alexa Fluor 488 | BioLegend | 301811 |
| CD86 | PE/Cy7 | BioLegend | 305422 |

**Table S10. Human MoDC differentiation**

## Supplementary Table 11

| **Target** | **Label** | **Company** | **Catalog Number** |
| --- | --- | --- | --- |
| LIVE/DEAD Fixable | Near-IR | ThermoFisher Scientific | L10119 |
| CD11c | APC | BioLegend | 337208 |

**Table S11. Human MoDC, AAC-HPV Uptake**

## Supplementary Table 12

| **Target** | **Label** | **Company** | **Catalog Number** |
| --- | --- | --- | --- |
| CD80 | Brilliant Violet 421 | BioLegend | 305222 |
| HLA-DR | Brilliant Violet 605 | BioLegend | 307640 |
| LIVE/DEAD Fixable | Near-IR | ThermoFisher Scientific | L10119 |
| CD11c | Alexa Fluor 488 | BioLegend | 301618 |
| CD40 | PE | BioLegend | 313006 |
| CD86 | PE/Cy7 | BioLegend | 305422 |

**Table S12. Human MoDC maturation by poly I:C**

## Supplementary Table 13

| **Target** | **Label** | **Company** | **Catalog Number** |
| --- | --- | --- | --- |
| IFNγ | Brilliant Violet 421 | BioLegend | 505830 |
| CD4 | Brilliant Violet 510 | BioLegend | 100559 |
| CD44 | Brilliant Violet 605 | BioLegend | 103047 |
| CD45 | Brilliant Violet 711 | BioLegend | 103147 |
| LIVE/DEAD Fixable | Near-IR | ThermoFisher Scientific | L10119 |
| TNFα | APC | BioLegend | 506308 |
| IL-2 | Alexa Fluor 488 | BioLegend | 503813 |
| CD8a | PerCp-Cy5.5 | BioLegend | 100734 |
| Granzyme B | PE | eBioscience | 12-8898-82 |
| B220 | PE/Cy7 | BioLegend | 103222 |
| CD11b | PE/Cy7 | BioLegend | 101216 |

**Table S13. TC-1 TIL/spleen ICS**

## Supplementary Table 14

| **Target** | **Label** | **Company** | **Catalog Number** |
| --- | --- | --- | --- |
| CD366 (Tim-3) | Brilliant Violet 421 | BioLegend | 119723 |
| CD45 | Brilliant Violet 711 | BioLegend | 103147 |
| LIVE/DEAD Fixable | Near-IR | ThermoFisher Scientific | L10119 |
| CD279 (PD-1) | APC | BioLegend | 135210 |
| B220 | FITC | BioLegend | 103206 |
| CD11b | FITC | BioLegend | 101206 |
| CD8a | PerCP-Cy5.5 | BioLegend | 100734 |
| CD4 | PE-Cy7 | BioLegend | 100422 |
| E7 Tetramer (H-2Db HPV16 E7_49-57_, RAHYNIVTF) | PE | MBL International | TB-5008-1 |

**Table S14. TC-1 TIL/spleen tetramer staining**

## Supplementary Table 15

| **Target** | **Label** | **Company** | **Catalog Number** |
| --- | --- | --- | --- |
| Ki-67 | Brilliant Violet 421 | BD Bioscience | 562899 |
| CD25 | Brilliant Violet 605 | Biolegend | 102036 |
| CD45 | Brilliant Violet 711 | Biolegend | 103147 |
| B220 | FITC | Biolegend | 103206 |
| CD11b | FITC | Biolegend | 101206 |
| NK1.1 | Alexa Fluor 700 | Biolegend | 108730 |
| LIVE/DEAD Fixable | Near-IR | ThermoFisher Scientific | L10119 |
| Foxp3 | APC | Thermo Fisher Scientific | 17-5773-82 |
| CD8a | PerCp-Cy5.5 | Biolegend | 100734 |
| CD4 | PE/Cy7 | Biolegend | 100422 |

**Table S15. TC-1 TIL/spleen Tregs staining**
